# *In vitro* eradication of abasic site-mediated DNA–peptide/protein cross-links by *Escherichia coli* long-patch base excision repair

**DOI:** 10.1016/j.jbc.2022.102055

**Published:** 2022-05-20

**Authors:** Cameron Bryan, Xiaoying Wei, Zhishuo Wang, Kun Yang

**Affiliations:** 1 Division of Chemical Biology and Medicinal Chemistry, College of Pharmacy, The University of Texas at Austin, Austin, Texas, USA; 2 Department of Molecular Biosciences, The University of Texas at Austin, Austin, Texas, USA

**Keywords:** DNA damage, DNA repair, base excision repair, abasic site, DNA–protein cross-link, endonuclease IV, DNA polymerase I, AP site, apurinic/apyrimidinic or abasic site, APE1, AP endonuclease 1, BER, base excision repair, DPC, DNA–protein cross-link, dU, 2′-deoxyuracil, Endo IV, endonuclease IV, Exo III, exonuclease III, GAPDH, glyceraldehyde 3-phosphate dehydrogenase, HR, homologous recombination, MALDI-TOF, matrix-assisted laser desorption ionization time-of-flight (mass spectrometry), NER, nucleotide excision repair, PAGE, polyacrylamide gel electrophoresis, Pol I, DNA polymerase I, UDG, uracil DNA glycosylase

## Abstract

Apurinic/apyrimidinic (AP or abasic) sites are among the most abundant DNA lesions. Numerous proteins within different organisms ranging from bacteria to human have been demonstrated to react with AP sites to form covalent Schiff base DNA–protein cross-links (DPCs). These DPCs are unstable due to their spontaneous hydrolysis, but the half-lives of these cross-links can be as long as several hours. Such long-lived DPCs are extremely toxic due to their large sizes, which physically block DNA replication. Therefore, these adducts must be promptly eradicated to maintain genome integrity. Herein, we used *in vitro* reconstitution experiments with chemically synthesized, stable, and site-specific Schiff base AP-peptide/protein cross-link analogs to demonstrate for the first time that this type of DPC can be repaired by *Escherichia coli* (*E. coli*) long-patch base excision repair. We demonstrated that the repair process requires a minimum of three enzymes and five consecutive steps, including: (1) 5′-DNA strand incision of the DPC by endonuclease IV; (2 to 4) strand-displacement DNA synthesis, removal of the 5′-deoxyribose phosphate-peptide/protein adduct-containing flap, and gap-filling DNA synthesis by DNA polymerase I; and (5) strand ligation by a ligase. We further demonstrated that endonuclease IV plays a major role in incising an AP-peptide cross-link within *E. coli* cell extracts. We also report that eradicating model AP-protein (11.2–36.1 kDa) DPCs is less efficient than that of an AP-peptide_10mer_ cross-link, supporting the emerging model that proteolysis is likely required for efficient DPC repair.

An apurinic/apyrimidinic (AP, abasic, Fig. 1*A*) site is one of the most abundant DNA lesions that is produced from the spontaneous or enzymatic hydrolysis of the glycosidic bond. Under typical conditions, ∼10,000 AP sites are produced per cell per day (1). The number increases significantly upon the exposure of DNA to alkylating agents (2). AP sites are cytotoxic due to their abilities to impede DNA replication and transcription (3, 4). AP sites at the stalled replication forks can be bypassed by translesion DNA synthesis polymerases (5). The lesion bypass DNA synthesis is highly mutagenic since the AP site is a noninstructional lesion (6). AP sites are repaired by base excision repair (BER) and nucleotide excision repair (NER), and the former is the major pathway (7, 8). BER of the AP site is conserved and includes short-patch and long-patch repair, and both pathways involve four core steps: strand incision, end processing, gap-filling DNA synthesis, and strand ligation (Fig. 2) (9, 10, 11).

**Figure 1:**
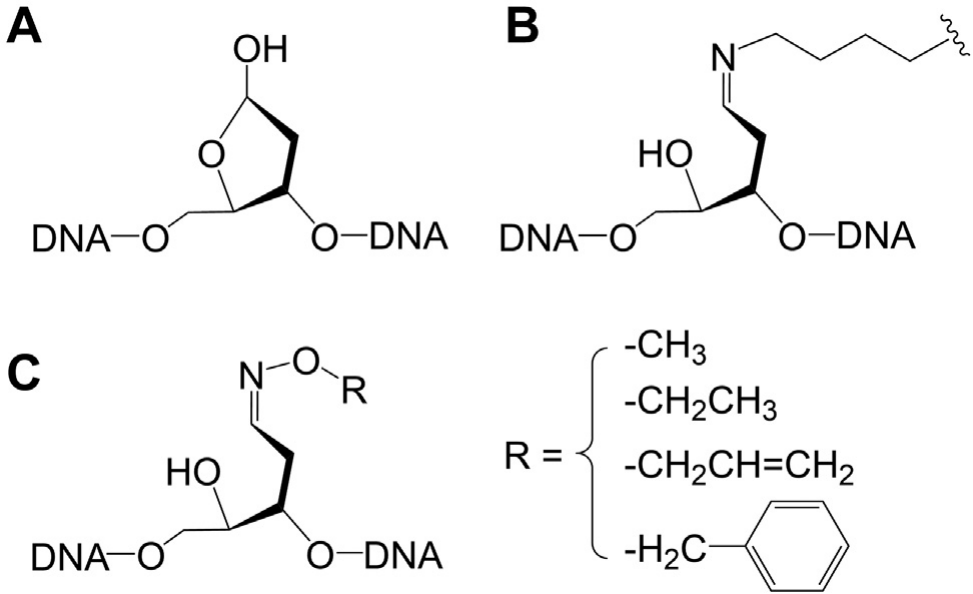
**Structure of AP siteand modified AP sites.***A*, AP site. *B*, Schiff base-linked AP-peptide/protein cross-link. *C,* alkylhydroxylamine-conjugated AP site. AP site, apurinic/apyrimidinic or abasic site.

**Figure 2:**
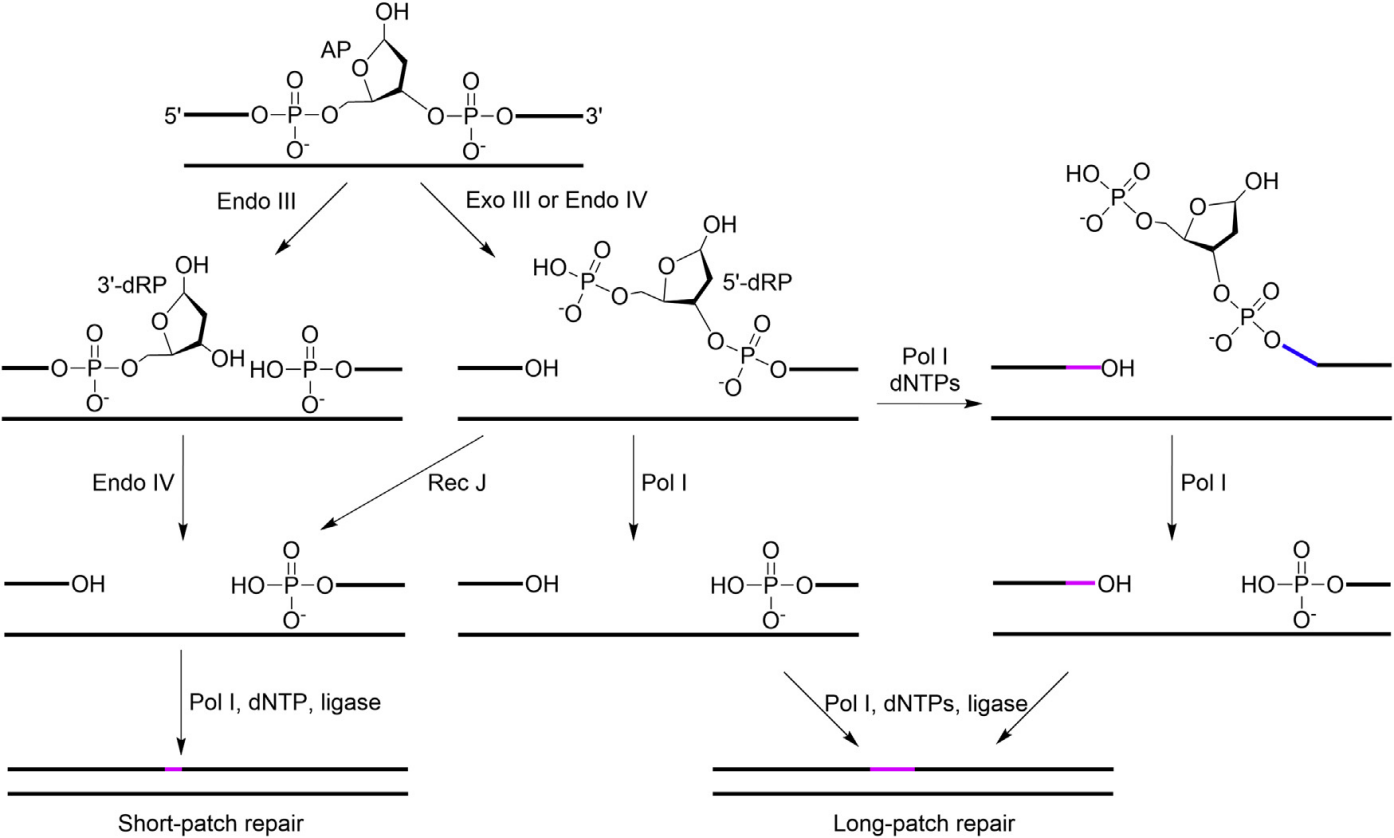
**Base excision repair of an AP site in *E. coli*** (9, 10, 11)**.** AP site, apurinic/apyrimidinic or abasic site.

If left unrepaired, AP sites can react with the N terminal or lysine side chain amines in peptides (12) and numerous proteins (13, 14, 15, 16, 17, 18, 19, 20, 21, 22) to form covalent Schiff base DNA–peptide/protein cross-links (DPCs, Fig. 1*B*). Schiff base AP-protein DPC formation has been demonstrated *in vitro* using AP site-containing DNA and recombinant proteins (13, 14, 15, 16, 17, 18, 19, 20, 21, 22) or *Escherichia coli* (*E. coli*) and yeast cell extracts (23) and also in human cells with an abundance of ∼1500/cell under normal conditions (24, 25). Schiff base DPCs are unstable due to the spontaneous hydrolysis; however, their half-lives can be as long as several hours under physiological pH and temperature (14, 26). A recent mass spectrometry study captured several Schiff base-linked 2′-deoxyribose-peptide adducts following digestion of the DPCs that were isolated from methyl methanesulfonate–treated HeLa cells, indicating that some of the Schiff base AP-peptide/protein cross-links are quite stable (24). The bulky Schiff base AP-protein DPCs completely block the DNA synthesis of various *E. coli* and human DNA polymerases (27). Therefore, they need to be promptly removed to maintain the genome integrity. Reduced Schiff base AP-protein adducts have been shown to be eradicated by recombinant *E. coli* UvrABC endonuclease, and smaller peptide adducts are more favorable substrates (28, 29). These DPCs have also been demonstrated to be repaired by NER in human cells, and homologous recombination (HR) in human mitochondria, possibly coupled with proteolysis (30, 31, 32). Whether other repair pathway(s) exists remains elusive.

Herein, we wish to report our *in vitro* evidence that Schiff base AP-peptide/protein cross-links can be repaired by *E. coli* long-patch BER. With chemically synthesized, site-specific, and stable AP-peptide_10mer_ adducts that mimic the Schiff base AP-peptide/protein DPC, we first stepwisely reconstituted the DPC repair *in vitro* and demonstrated that AP-peptide_10mer_ cross-links can be repaired within five continuous steps involving the cooperation of 3 *E. coli* enzymes: endonuclease IV (Endo IV), DNA polymerase I (Pol I), and ligase. Using cell extracts prepared from DNA repair–deficient *E. coli* strains, we then demonstrated that Endo IV plays a major role in incising the AP-peptide_10mer_ cross-link. Finally, we found that eradicating reduced Schiff base AP-protein (11.2–36.1 kDa) DPCs is less efficient than that of an AP-peptide_10mer_ adduct, which agrees with the emerging model that proteolysis is required for efficient DPC repair. To our knowledge, this is the first time to reveal that DPCs can be repaired by *E. coli* long-patch BER. We envision that this novel DPC repair pathway is conserved in prokaryotes and lower eukaryotes.

## Results

### Endo IV, but not exonuclease III, incises the 5′-side of an AP-peptide_10mer_ cross-link

The goal of this study is to investigate whether Schiff base AP-protein DPCs can be repaired by other pathways other than NER and HR. *E. coli* exonuclease III (Exo III) and Endo IV, the enzymes responsible for 5′-strand incision of the AP site (Fig. 2), have been demonstrated to be able to incise the alkylhydroxylamine-conjugated AP sites (Fig. 1*C*) (33, 34) that are structurally similar to the Schiff base AP-protein DPCs (Fig. 1*B*). Inspired by this, we asked whether Exo III and/or Endo IV can incise the Schiff base AP-peptide/protein cross-links and the generated 5′-dRP-peptide/protein cross-links can then be removed similarly to 5′-dRP (Fig. 2).

**Figure 3:**
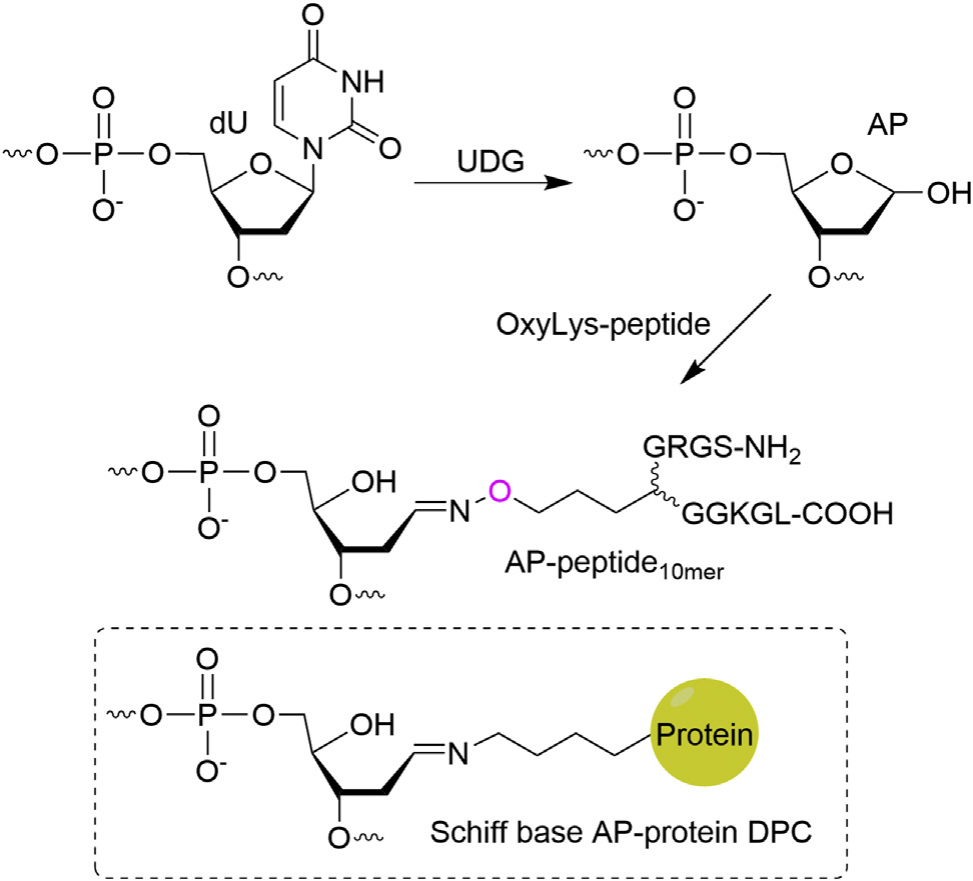
**Synthesis of an AP-peptide**_**10mer**_**cross-link by oxime ligation.** AP site, apurinic/apyrimidinic or abasic site; DPC, DNA–protein cross-link; UDG, uracil DNA glycosylase.

To address the above question *in vitro*, a stable substrate is needed. The Schiff base is unstable upon heating at high temperatures (*e.g.,* 90 °C) which is often required to dehybridize the duplex DNA prior to the urea-PAGE analysis. Reductive amination that uses NaBH_3_CN or NaBH_4_ to reduce the Schiff base linkage has been utilized to prepare stable AP-peptide/protein cross-links (28, 29). Herein, we reported a new chemical approach to prepare a stable and site-specific Schiff base AP-peptide cross-link analog through a bioorthogonal oxime ligation involving reacting the AP site with an aminooxylysine (OxyLys)-containing peptide (Fig. 3). Compared to a Schiff base AP-protein DPC, the linkage prepared through oxime ligation is stable and has only one difference that the ε-carbon of the lysine residue is replaced by an oxygen atom. Specifically, a 2′-deoxyuracil (dU)-containing oligo (Table 1, O2) with a 6-carboxyfluorescein (6-FAM) at the 5′-terminus was treated by *E. coli* uracil-DNA glycosylase (UDG) to generate an AP site. A 10-mer model peptide (918.1 Da) derived from human histone H4_1-10_ (NH_2_-SGRGK_5_GGKGL-COOH) with the replacement of lysine 5 by an OxyLys was synthesized through solid-phase peptide synthesis (35). Conjugation of the AP site to OxyLys-peptide_10mer_ yielded AP-peptide_10mer_ (Table 1, P1; Fig. S1*A*). The adduct was isolated, and the purity was verified by urea-PAGE (Fig. S1*B*). The correct molecular weight was confirmed by matrix-assisted laser desorption ionization time-of-flight (MALDI-TOF) mass spectrometry (Fig. S1*C*). The formation of a desired oxime linkage instead of an imine from the N terminal or lysine side chain amine was confirmed by the observation (Fig. S1*D*) that the adduct was stable to heating (70 °C for 1 h or 90 °C for 10 min) and NaOH treatment (0.1 M, 37 °C, 1 h) as the latter is unstable and will decompose under these conditions (36). AP-peptide_10mer_ with 6-FAM at the 3′-terminus (Table 1, P2) was synthesized and characterized similarly (Fig. S2).

**Table 1:** Oligos and DNA–peptide cross-links used in this study

O1	5′-ATTGAGCGGCCTCGGCACCGGGATTCTGAT-3′ (competitor for reaction quenching)
O2	5′-6-FAM-CGAGATCTGAGTCCGGUAGCGCTAGCG-3′ (Prepare P1)
O3	5′-CGCTAGCGCTACCGGACTCAGATCTCG-3′ (Fig. 4*A*)
O4	5′-GGAGTCGCTTUCGCAAAGCTTGAGCTC-3′-6-FAM (Prepare P2 and P4, Fig. S5)
O5	5′-GAGCTCAAGCTTTGCGAAAGCGACTCC-3′ (Fig. 4*B*)
O6	5′-Cy5-TGCAGAATTCGGAGTCGCTTUCGCAAAGCTTGAGC(FdT)C-3′ (Prepare P3 and AP-H4 DPC)
O7	5′-GAGCTCAAGCTTTGCGAAAGCGACTCCGAATTCTGCA-3′ (Figs. 5, 6 and 11)
O8	5′-TGCAGAATTCGGAGTCGCTT-3′ (Fig. 6)
O9	5′-GAGCTCAAGCTTTGCGAAGCGACTCCGAATTCTGCA-3′ (Fig. 6)
O10	5′-GCCGGCGCGCUACGCAAAGCTTGAGCTC-3′-6-FAM (Prepare P5)
O11	5′-GAGCTCAAGCTTTGCGTAGCGCGCCGGC-3′ (Prepare P5)
O12	5′-GCCGGCGCGCUAAACGCAAAGCTTGAGCTC-3′-6-FAM (Prepare P6)
O13	5′-GAGCTCAAGCTTTGCGTTTAGCGCGCCGGC-3′ (Prepare P6)
O14	5′-phosphate-CAAAGCTTGAGCTC-3′-6-FAM (Fig. 6, marker)
O15	5′-phosphate-AAAGCTTGAGCTC-3′-6-FAM (Fig. 6, marker)
O16	5′-phosphate-AGCTTGAGCTC-3′-6-FAM (Fig. 6, marker)
O17	5′-GCGCAAAGCTTGAGCUCGAGATCTGAGTCCGGT-3′ (Prepare P7)
O18	5′-ACCGGACTCAGATCTCGAGCTCAAGCTTTGCGC-3′ (Gap plasmid construction)
O19	5′-6-FAM-CGAGATCTGAGTCCGGUAGCGCTAGCGGATCTGACGGTTCAC-3′ (Prepare AP-protein DPCs)
O20	5′-GTGAACCGTCAGATCCGCTAGCGCTACCGGACTCAGATCTCG-3′ (Fig. 10)
P1	5′-6-FAM-CGAGATCTGAGTCCGG(AP-peptide_10mer_)AGCGCTAGCG-3′ (Fig. 4*A*)
P2	5′-GGAGTCGCTT(AP-peptide_10mer_)CGCAAAGCTTGAGCTC-3′-6-FAM (Figs. 4*B* and S5)
P3	5′-Cy5-TGCAGAATTCGGAGTCGCTT(AP-peptide_10mer_)CGCAAAGCTTGAGC(FdT)C-3′ (Fig. 5)
P4	5′-dRP-peptide_10mer_-CGCAAAGCTTGAGCTC-3′-6-FAM (Fig. 6)
P5	5′-dRP-peptide_10mer_-ACGCAAAGCTTGAGCTC-3′-6-FAM (Fig. 6)
P6	5′-dRP-peptide_10mer_-AAACGCAAAGCTTGAGCTC-3′-6-FAM (Fig. 6)
P7	5′-phosphate-GCGCAAAGCTTGAGC(AP-peptide_10mer_)CGAGATCTGAGTCCGGT-3′ (Prepare pHha10-AP-peptide_10mer_)

**Figure 4:**
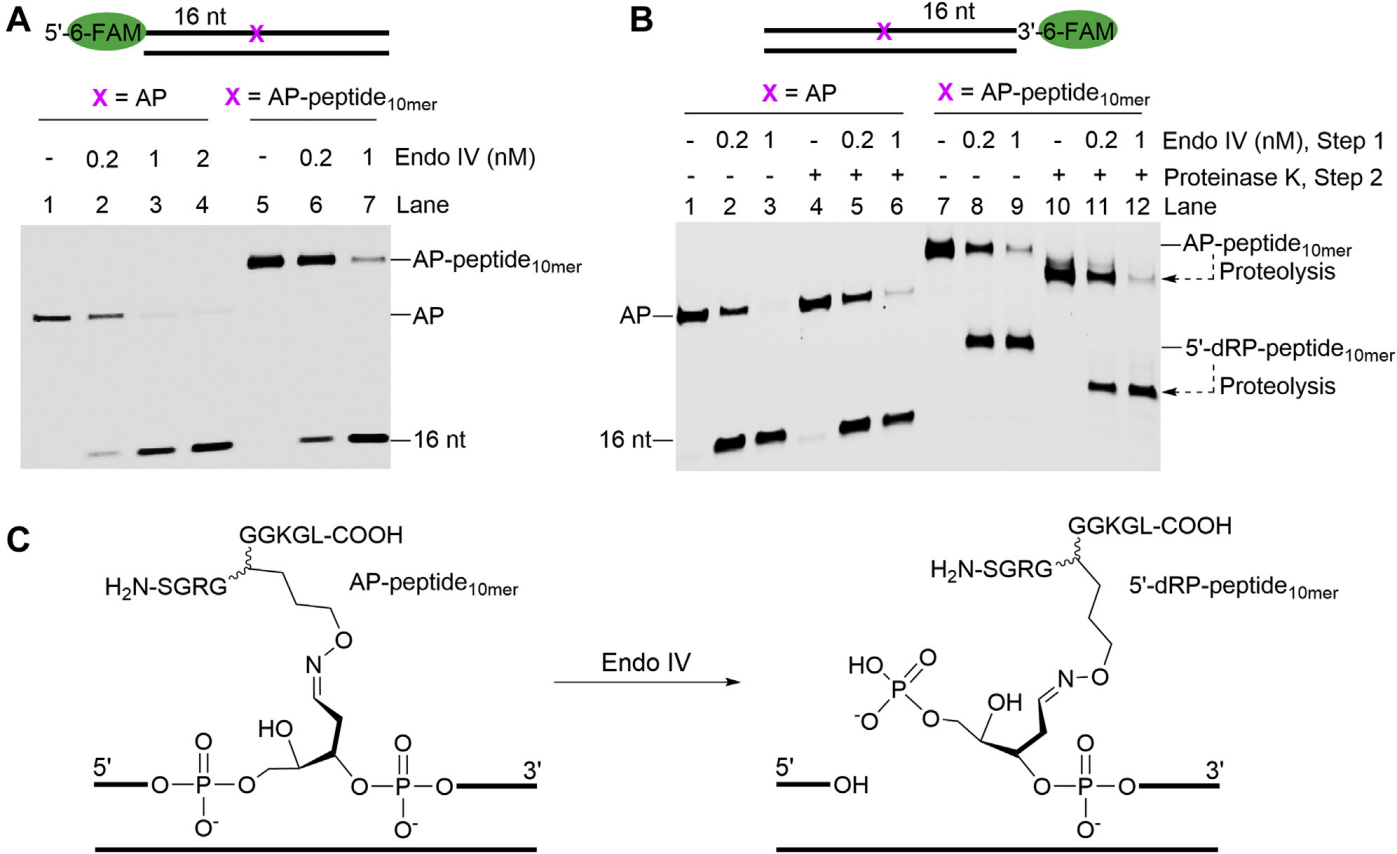
**Endo IV incises AP-peptide**_**10mer**_. *A*–*B*, representative 20% urea-PAGE gels showing the strand incision of an AP site (20 nM) or AP-peptide_10mer_ (20 nM) by Endo IV with indicated enzyme concentrations at 37 ^°^C for 30 min. The AP site was prepared from oligo O2 (*A*) or O4 (*B*). The AP-peptide_10mer_ is P1 (*A*) or P2 (*B*). The oligos and DNA–peptide adducts were visualized by using the fluorescence of 6-FAM. *C*, illustration of incision of AP-peptide_10mer_ by Endo IV, which yields a 3′-OH and 5′-dRP-peptide_10mer_. AP site, apurinic/apyrimidinic or abasic site; Endo IV, endonuclease IV.

With AP-peptide_10mer_ in hands, we investigated whether it can be incised by Exo III and/or Endo IV. Specifically, the AP-peptide_10mer_ was first hybridized to a complementary strand and then treated by Exo III or Endo IV, followed by urea-PAGE analysis and visualization using the fluorescence of 6-FAM. As shown in Fig. S3, Exo III incised the AP site but not the AP-peptide_10mer._ The faster migrating bands were attributed to the 3′ to 5′ exonuclease products. On the contrary, Endo IV incised the AP-peptide_10mer_ (Fig. 4). When the 6-FAM is conjugated at the 5′-terminus, the incised AP-peptide_10mer_ migrated the same to the cleaved AP site (Fig. 4*A*, Lanes 2–4 *versus* 6–7). When the 3′-terminus is conjugated with a 6-FAM, the incised adduct migrated much slower than the cleaved AP site (Fig. 4*B*, Lanes 2–3 *versus* 8–9), which is ascribed to the conjugation of the peptide_10mer_. This is supported by the observation that an additional treatment with proteinase K generated a faster migrating product (Fig. 4*B*, Lanes 8–9 *versus* 11–12), and it migrated slightly slower than the incised AP site (Fig. 4*B*, Lanes 5–6 *versus* 11–12). Based on these results and the known catalytic mechanism of Endo IV (37), we conclude that Endo IV, but not Exo III, can incise the 5′-side of the AP-peptide_10mer_, yielding a 3′-OH and a 5′-dRP-peptide_10mer_ (Fig. 4*C*).

Next, we determined the steady-state kinetic constants (Table 2 and Fig. S4) of AP-peptide_10mer_ incision by Endo IV. Similar to the previously reported catalytic efficiency (*k*_cat_/*K*_m_ = 0.17–0.36 min^−1^ nM^−1^) (38, 39), Endo IV incises the AP site with the *k*_cat_/*K*_m_ of ∼0.33 min^−1^ nM^−1^ under our conditions. Notably, compared to the AP site, the catalytic efficiency (*k*_cat_/*K*_m_ = 0.21 ± 0.01 min^−1^ nM^−1^) of incising AP-peptide_10mer_ adduct is only 1.6-fold lower, and this is due to a smaller *k*_cat_. To our knowledge, this is the first time to demonstrate that Endo IV can efficiently incise the DNA strand at a bulky AP-peptide_10mer_ adduct.

**Table 2:** Steady-state kinetic constants of incising an AP site and AP-peptide_10mer_ by Endo IV.

DNA lesion	*K*_m_ (nM)^a^	*k*_cat_ (min^−1^)^a^	*k*_cat_/*K*_*m*_ (min^−1^ nM^−1^)^a^
AP	57.8 ± 5.3	19.5 ± 3.5	0.33 ± 0.03
AP-peptide_10mer_	59.9 ± 2.6	12.1 ± 0.3	0.21 ± 0.01

a Values are the average ± standard deviation of three independent experiments.

### Pol I removes 5′-dRP-peptide_10mer_ following strand-displacement DNA synthesis

Having demonstrated that Endo IV efficiently incises AP-peptide_10mer_, we then asked whether the resulting 5′-dRP-peptide_10mer_ can be removed similarly to 5′-dRP (Fig. 2), which is required before the strand ligation. Pol I is a multifunctional enzyme that functions as a DNA polymerase, 3′ to 5′ exonuclease, or 5′-flap endonuclease (9, 40). Similar to the previous observation (9), after incision of the AP site by Endo IV, Pol I removed 5′-dRP mainly with two nucleotides (Fig. S5, Lane 3). However, following Endo IV incision of the AP-peptide_10mer_, removal of 5′-dRP-peptide_10mer_ was barely detected even when a 10-fold higher concentration of Pol I was used (Fig. S5, Lane 12). This is possibly due to the steric hindrance of the cross-linked 10-mer peptide. The 5′-dRP has also been reported to be excised by *E. coli* RecJ, a 5′ to 3′ exonuclease (10). However, such activity is controversial as it was not observed by Lloyd *et al*. (41). Under our conditions, we did not observe the removal of 5′-dRP-peptide_10mer_ by RecJ (data not shown).

**Figure 5:**
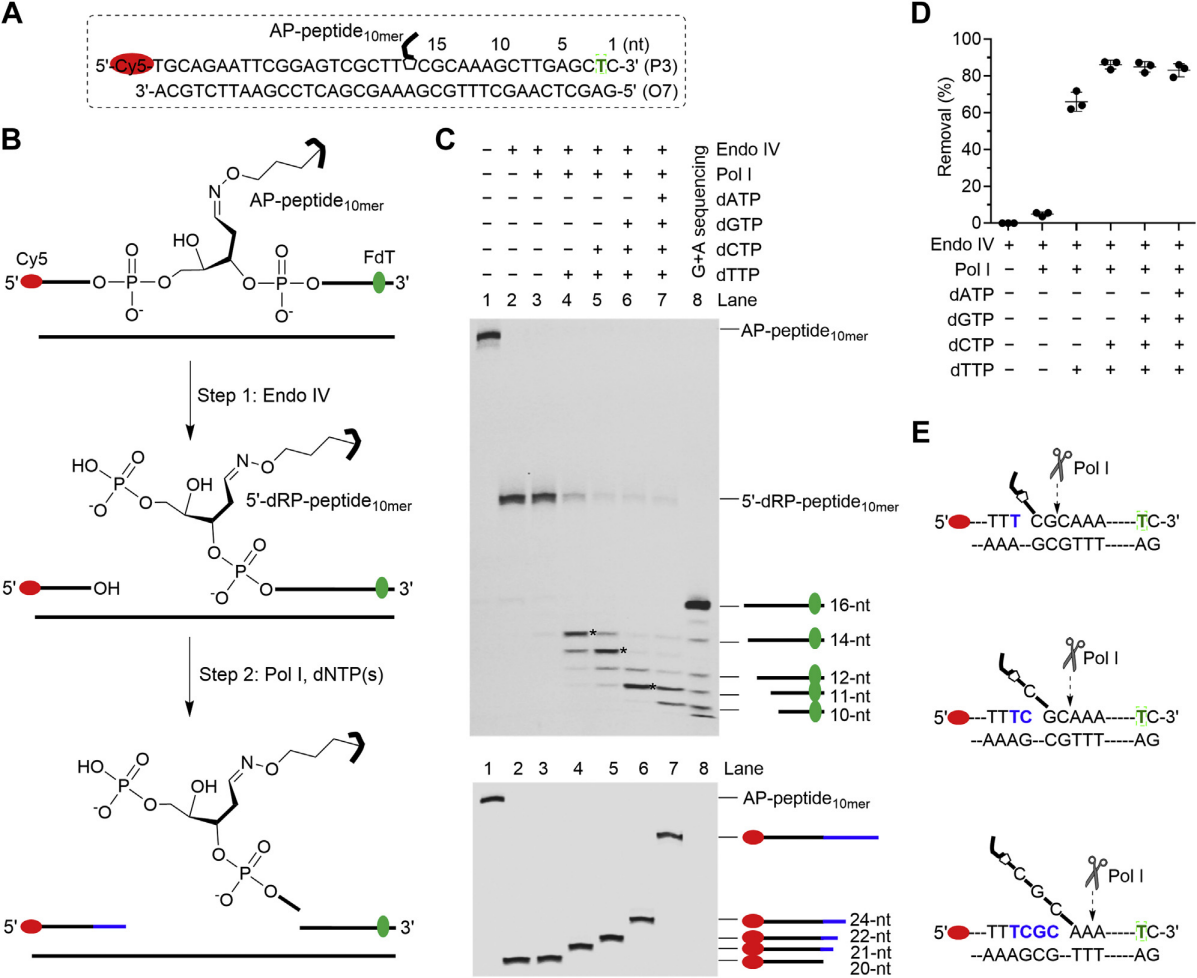
**Pol I removes 5′-dRP-peptide**_**10mer**_**following strand-displacement DNA synthesis**. *A*, the nucleotide sequence of duplex DNA containing AP-peptide_10mer_. A Cy5 is at the 5′-terminus, and a FdT is at the second position from the 3′-terminus. The numbers above the nucleotide sequence indicate the lengths from the 3′-terminus. *B*, a scheme showing the procedures of investigating the strand-displacement DNA synthesis and 5′-dRP-peptide_10mer_ removal by Pol I following the Endo IV-induced strand incision. *C*, a representative 20% urea-PAGE gel showing the removal of 5′-dRP-peptide_10mer_ (20 nM, top, FdT) and strand-displacement DNA synthesis (bottom, Cy5) by Pol I (4 nM) in the presence of different dNTPs (2 μM). The reactions were carried out at 37 ^°^C for 30 min. The *asterisks* (top) indicate the predominant excision products. *D*, a scatter plot with the mean and standard deviation showing the efficiency of 5′-dRP-peptide_10mer_ removal by Pol I from reactions in C (top). The data are from three independent experiments. *E*, the predominant excision sites of Pol I following the strand-displacement DNA synthesis that were determined from the results in C (top, Lanes 4–6). AP, apurinic/apyrimidinic or abasic; Endo IV, endonuclease IV; FdT, fluorescein dT; Pol I, DNA polymerase I.

Next, we asked whether Pol I can perform the strand-displacement DNA synthesis and then remove the 5′-dRP-peptide_10mer_-containing DNA flap following Endo IV-induced strand incision. To address this question, we synthesized an AP-peptide_10mer_ cross-link (Table 1, P3; Fig. S6) bearing a Cy5 fluorophore at the 5′-terminus for detecting the strand-displacement DNA synthesis products, and a fluorescein dT (FdT) at the second position from the 3′-terminus to detect the 5′-dRP-peptide_10mer_ removal. The AP-peptide_10mer_ was first hybridized to the complementary strand (Fig. 5*A*) and then completely incised by Endo IV, followed by addition of Pol I with an individual or a combination of dNTPs (Fig. 5*B*). The reaction products were analyzed by 20% urea-PAGE and visualized by using the fluorescence of Cy5 or FdT (Fig. 5*C*). Similar to the above results, very inefficient (∼4%) 5′-dRP-peptide_10mer_ removal was observed in the absence of dNTPs (Fig. 5*C*, top, Lane 3; Fig. 5*D*). Notably, approximately 60% of the adduct was removed when dTTP was added, and ∼80% of that was removed in the presence of dTTP + dCTP, dTTP + dCTP + dGTP, or a full set of dNTPs (Fig. 5*C*, top, Lanes 4–7; Fig. 5*D*). Under these conditions, 1-nt, 2-nt, 4-nt, and full-length strand-displacement DNA synthesis products were observed, respectively (Fig. 5*C*, bottom). Following 1-nt, 2-nt, or 4-nt of strand-displacement DNA synthesis, the predominant cleavage site of Pol I is at the second nucleotide after the junction between the single strand and duplex regions (Fig. 5*C*, top, Lanes 4–6; Fig. 5*E*). Based on these results, we conclude that (1) Pol I is able to perform the strand-displacement DNA synthesis when encountering a 5′-dRP-peptide_10mer_; (2) Pol I can subsequently remove a polynucleotide flap containing a 5′-dRP-peptide_10mer_, and the predominant excision site is at the second nucleotide after the single and double-strand junctions; (3) the minimal length of the strand-displacement DNA synthesis for Pol I to achieve the maximal 5′-dRP-peptide_10mer_ removal efficiency is two.

**Figure 6:**
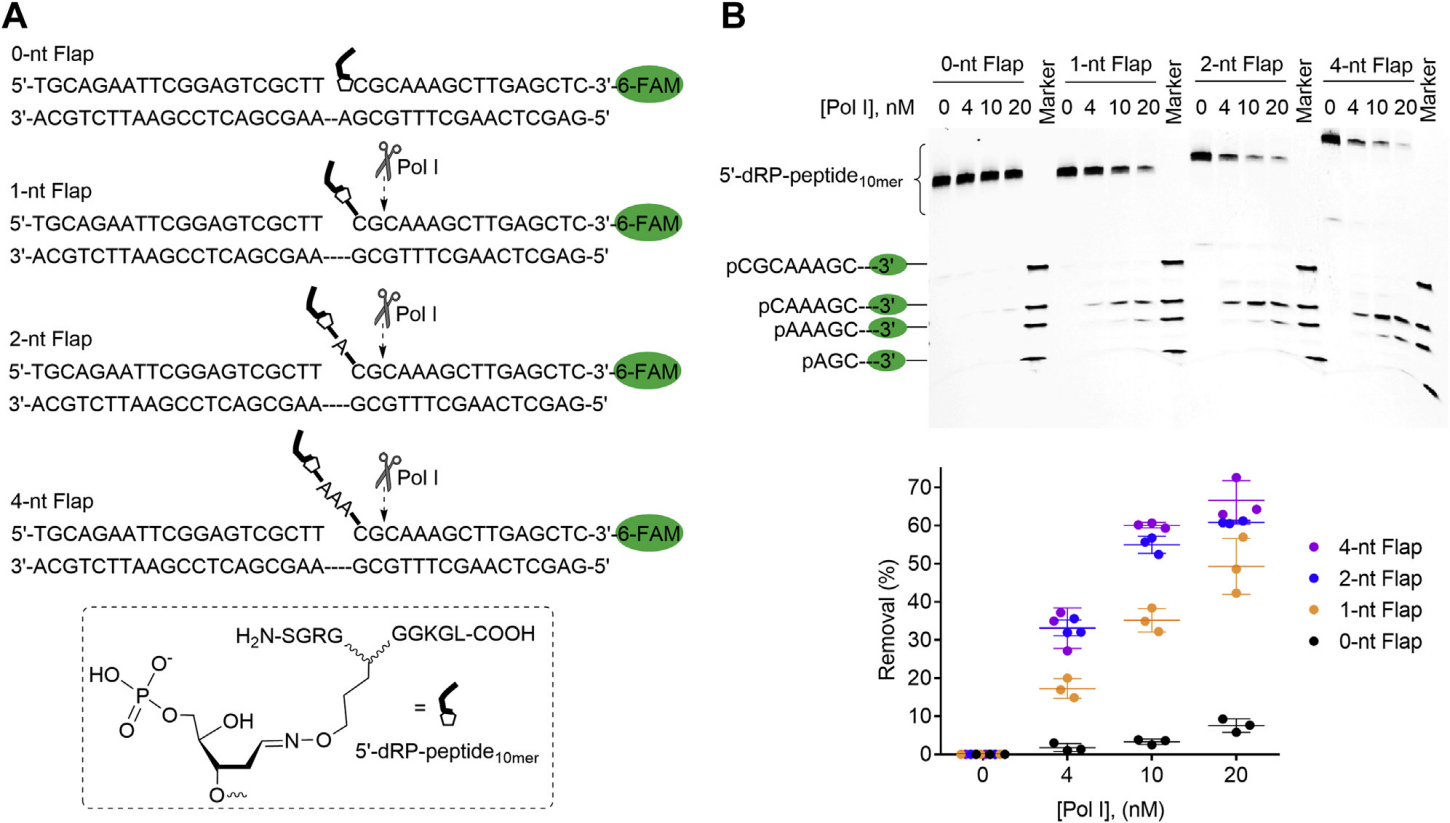
**Removal of independently generated 5′-dRP-peptide**_**10mer**_**-containing flaps by Pol I**. *A*, nucleotide sequences of nicked DNA containing independently generated 5′-dRP-peptide_10mer_ within different lengths of flaps. The *arrows* indicate the predominant incision sites by Pol I that were determined from the results in B. *B*, top, a representative 20% urea-PAGE gel showing the removal of 5′-dRP-peptide_10mer_ (20 nM) by Pol I (0–20 nM) at 37 ^°^C for 30 min. The oligos and DNA–peptide adducts were visualized by using the fluorescence of 6-FAM. Bottom, a scatter plot with the mean and standard deviation showing the efficiency of 5′-dRP-peptide_10mer_ removal by Pol I as a function of Pol I concentration. The data are from three independent experiments. Pol I, DNA polymerase I.

**Figure 7:**
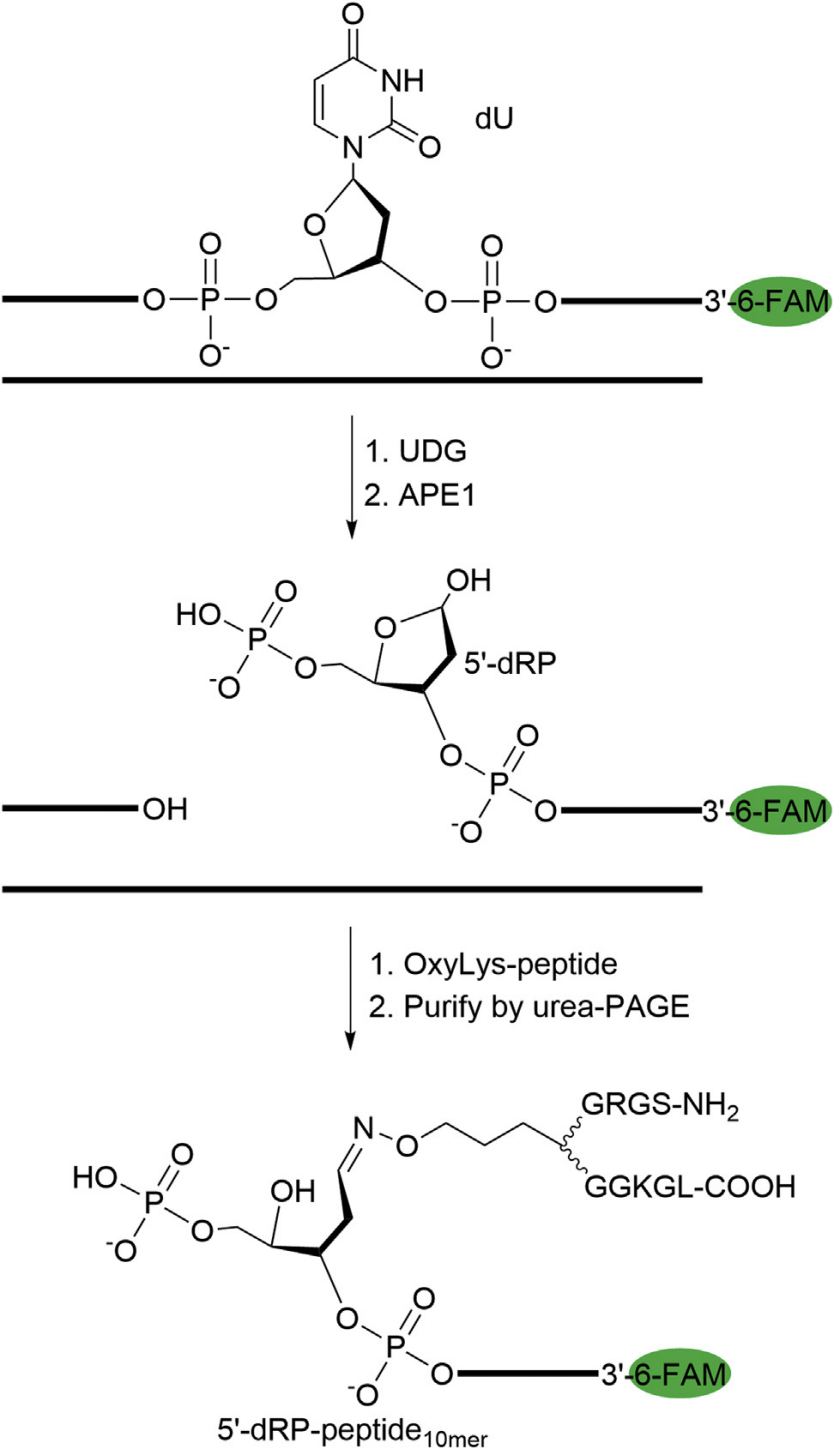
**Synthesis of a 5′-dRP-peptide**_**10mer**_**cross-link by oxime ligation.** APE1, AP endonuclease 1; UDG, uracil DNA glycosylase.

To further support the above conclusions, four nicked DNA substrates bearing independently synthesized 5′-dRP-peptide_10mer_ were prepared to mimic different lengths (0-nt, 1-nt, 2-nt, 4-nt) of strand-displacement DNA synthesis products (Fig. 6*A*). To synthesize the 5′-dRP-peptide_10mer_, a dU-containing oligo with 6-FAM at the 3′-terminus was hybridized to a complementary strand and then sequentially treated by UDG and human AP endonuclease 1 (APE1) to yield a 5′-dRP, followed by reacting with the 10-mer OxyLys-containing peptide (Fig. 7). The 5′-dRP-peptide_10mer_ adducts (Table 1, P4-6) were purified and characterized similarly to AP-peptide_10mer_ (Fig. S7). These adducts were then hybridized to form nicked DNA substrates (Fig. S8) that mimic the intermediates produced after the strand incision and strand-displacement DNA synthesis. The nicked DNA substrates were then incubated with increasing concentration of Pol I in the absence of dNTPs, followed by urea-PAGE analysis. As shown in Figure 6, agreeing with all above observations, efficient excision of 5′-dRP-peptide_10mer_ by Pol I was only observed when the adduct is within a flap; the highest efficiency was observed when the flap length is two or longer; and the predominant excision site is at the second nucleotide after the single and double-strand junctions.

### Reconstitution of E. coli long-patch BER of an AP-peptide_10mer_ cross-link

**Figure 8:**
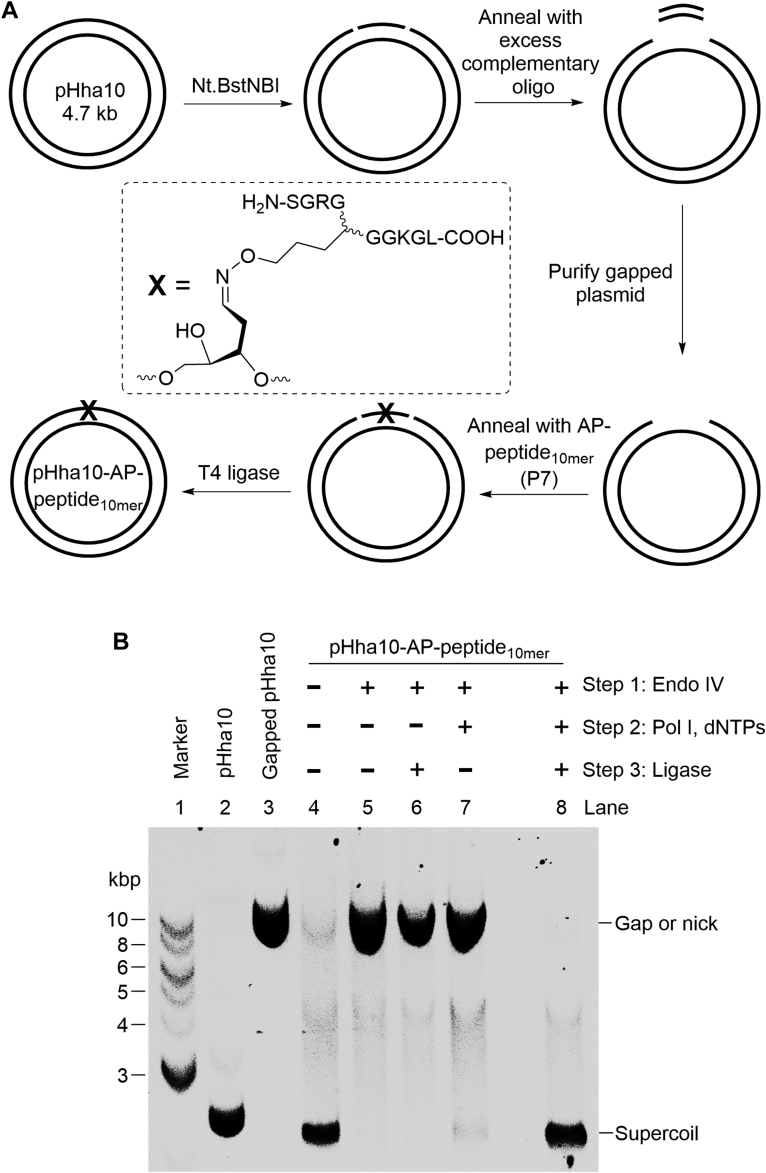
***In vitro* reconstitution of *E. coli* long-patch base excision repair of an AP-peptide**_**10mer**_**cross-link**. *A*, illustration of preparing a plasmid (pHha10-AP-peptide_10mer_) containing a site-specific AP-peptide_10mer_. *B*, a representative 1% agarose gel (prestained with SYBR Gold) showing the repair of AP-peptide_10mer_ within a plasmid. See the experimental section for detailed reaction conditions. AP, apurinic/apyrimidinic or abasic.

Coupled with the strand incision by Endo IV, Pol I removes the AP-peptide_10mer_ adduct, which yields a DNA gap that presumably can be filled and ligated, resulting in full repair of the AP-peptide_10mer_ cross-link. To affirm this, we reconstituted the AP-peptide_10mer_ repair within a plasmid containing a site-specific adduct. To construct the adduct-containing plasmid (pHha10-AP-peptide_10mer_, Fig. 8*A*), the plasmid pHha10 (42) was first nicked by Nt.BstNBI that cuts the plasmid twice within the same DNA strand. The excised oligo fragment was removed by hybridization to a complementary oligo, followed by repeated centrifugation with a 100 kDa cut-off Amicon centrifugal filter. The gapped plasmid was then ligated to a 5′-phosphorylated AP-peptide_10mer_ adduct (Table 1, P7; Fig. S9). The unligated gapped plasmid was removed by Exo III, and the remaining ligated plasmid was purified using the Qiagen PCR purification kit. The homogeneity of pHha10-AP-peptide_10mer_ was verified by the observation that the Endo IV treatment fully converted the ligated plasmid from supercoil to the one that migrated the same with the gapped pHha10 (Fig. 8*B*, Lanes 3–5). The Endo IV-treated adduct-containing plasmid was converted from a nick to supercoil, in which the adduct was removed, the gap was filled, and the strand was ligated, only when Pol I, dNTPs, and ligase were added (Fig. 8*B*, Lanes 5–8). Thus, we demonstrated that AP-peptide_10mer_ can be repaired by *E. coli* long-patch BER *in vitro* with a minimum of three enzymes, *i.e.,* Endo IV, Pol I, and ligase.

### Endo IV plays a major role in incising an AP-peptide_10mer_ cross-link within E. coli cell extracts

**Figure 9:**
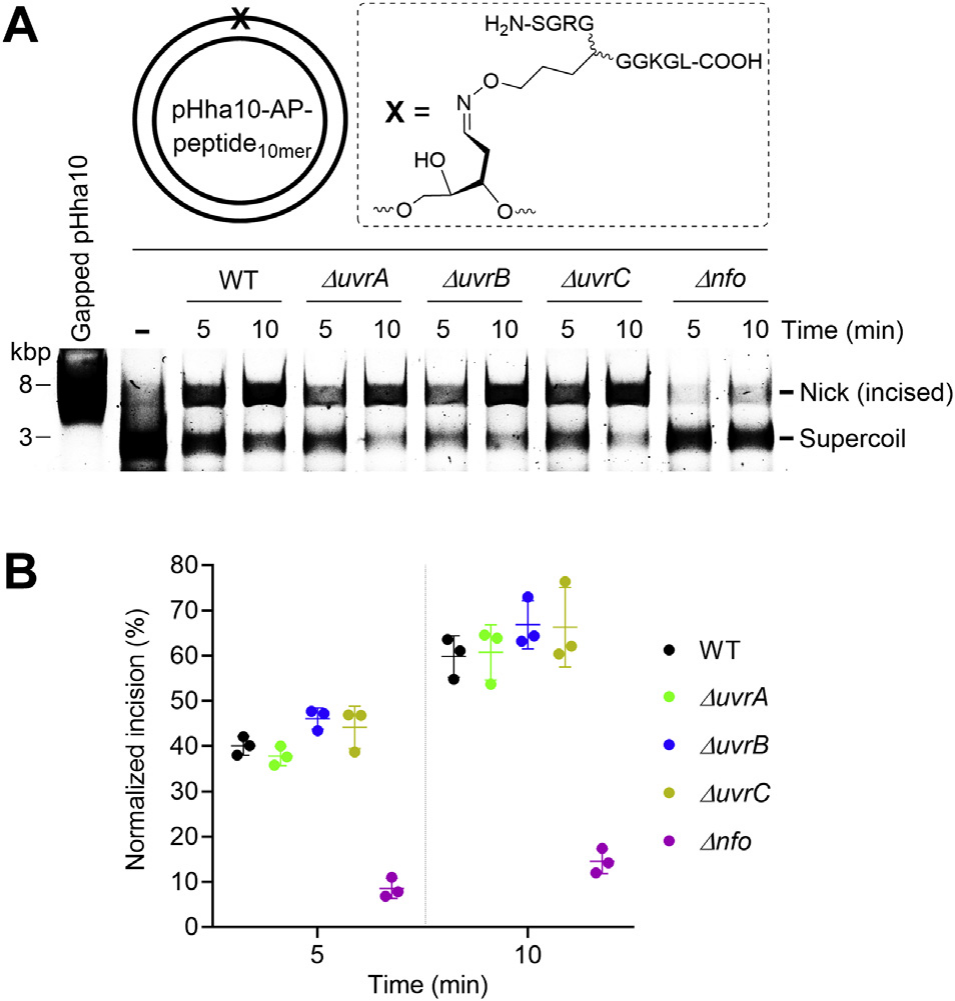
**Strand incision of pHha10-AP-peptide**_**10mer**_**by *E. coli* cell extracts**. *A*, a representative 1% agarose gel (prestained with SYBR Gold) showing the strand incision of pHha10-AP-peptide_10mer_ by WT or DNA repair–deficient *E. coli* cell extracts. See the experimental section for detailed reaction conditions. *B*, a scatter plot with the mean and standard deviation showing the strand incision efficiency of pHha10-AP-peptide_10mer_ by *E. coli* cell extracts as a function of time. The data are from three independent experiments. AP, apurinic/apyrimidinic or abasic.

To further confirm the role of Endo IV in incising AP-peptide adducts, pHha10-AP-peptide_10mer_ was incubated with wild-type (WT) or DNA repair-deficient *E. coli* cell extracts, followed by agarose gel analysis (Fig. 9*A*). As shown in Figure 9*B*, deletion of *uvrA*, *uvrB*, or *uvrC* that expresses the subunit of UvrABC endonuclease complex had little if any effect on the strand incision of AP-peptide_10mer_; however, deletion of *nfo* that expresses Endo IV reduced the efficiency by ∼75%. We did a control experiment to rule out the possibility that such difference was caused by improper preparation of the *nfo*-deficient cell extract. Specifically, using an uracil-containing plasmid as a substrate, we demonstrated that all cell extracts have similar activity of uracil-DNA glycosylase (Fig. S10). Based on these results, we conclude that under our conditions, Endo IV plays a major role in incising the AP-peptide_10mer_ within *E. coli* cell extracts.

### Endo IV incises reduced Schiff base AP-protein DPCs

**Figure 10:**
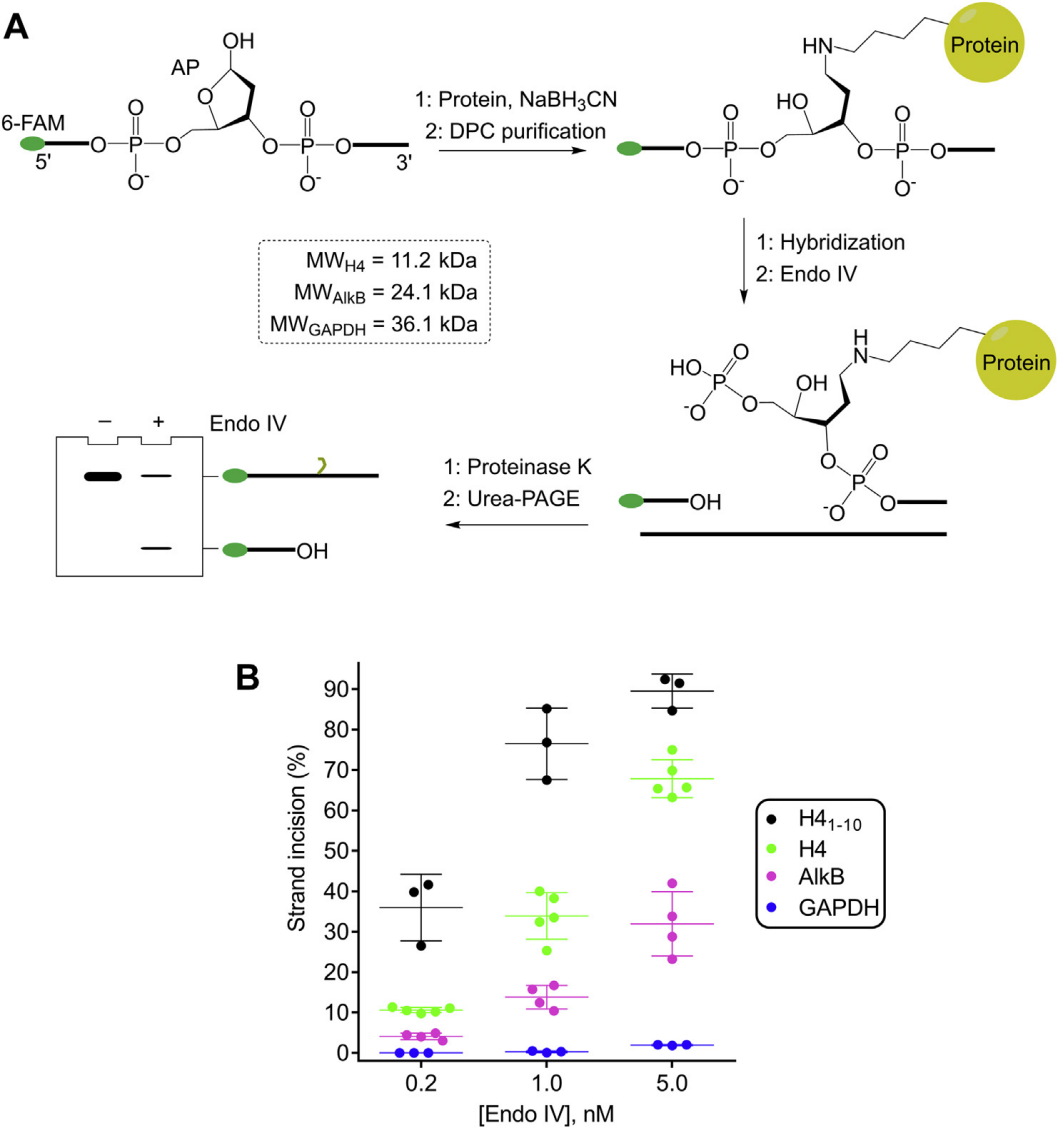
**Strand incision of model AP-protein DPCs by Endo IV**. *A*, a scheme showing the preparation of AP-protein DPCs by reductive amination and the product analysis following the strand incision by Endo IV. The AP site was prepared from oligo O19, and the complementary strand is oligo O20. All reaction samples were treated by proteinase K to facilitate the urea-PAGE analysis of the remaining uncleaved DPCs. *B*, a scatter plot with the mean and standard deviation showing the 5′-strand incision efficiency of AP-peptide_10mer_ (H4_1-10_, 20 nM) and AP-protein DPCs (20 nM) as a function of Endo IV concentration (0.2, 1, or 5 nM) at 37 ^o^C for 30 min. The data are from at least three independent experiments. AP site, apurinic/apyrimidinic or abasic site; DPC, DNA–protein cross-link; Endo IV, endonuclease IV.

Having demonstrated that an AP-peptide_10mer_ adduct can be repaired by *E. coli* long-patch BER, we then asked whether the larger AP-protein DPCs can be removed similarly. We first investigated the strand incision of AP-protein cross-links by Endo IV. Reductive amination was used to synthesize three stable model AP-protein cross-links with varied sizes (Fig. 10*A*). Specifically, human histone H4 (11.2 kDa), *E. coli* AlkB (24.1 kDa), and human glyceraldehyde 3-phosphate dehydrogenase (GAPDH) (36.1 kDa) were recombinantly purified and reacted with an AP site-containing single-strand oligo in the presence of NaBH_3_CN, which reduces and stabilizes the Schiff base DPCs. We chose these proteins because they have been demonstrated to be able to conjugate to an aldehyde-containing oligo through reductive amination (43). The reactions were analyzed by SDS-PAGE followed by DPC isolation (Fig. S11*A*). The desired AP-protein DPCs were determined by the observation that the proteinase K–treated DPCs migrated similarly with the uncleaved AP site-containing oligo (Fig. S11*B*). The DPCs within cleaved DNA that migrated similarly with the NaOH-incised AP site were formed *via* β-elimination (18). It should be noted that the DPC prepared through this approach is site-specific for DNA but not for proteins as both the N terminal and lysine side chain amines could react with the AP site. The purified AP-protein DPC was hybridized to the complementary strand and treated by increasing concentrations of Endo IV. To facilitate the urea-PAGE analysis of the remaining AP-protein DPCs, the reaction samples were treated by proteinase K following the Endo IV incision. Intriguingly, as shown in Figures 10*B* and S12, Endo IV incised AP-H4 and AP-AlkB DPCs although the efficiency is several times lower than that of the AP-peptide_10mer_. The reduced efficiency is likely due to the increased steric hindrance. This is further supported by the observation that conjugating a larger protein, GAPDH, to the AP site completely prevented the strand incision by Endo IV (Figs. 10*B* and S12). These results with model DPCs suggested that whether proteolysis is required for the strand incision by Endo IV largely depends on the size of the protein cross-linked to DNA, but proteolysis will facilitate the strand incision.

### Pol I excises the 5′-dRP-H4 DPC following strand-displacement DNA synthesis

**Figure 11:**
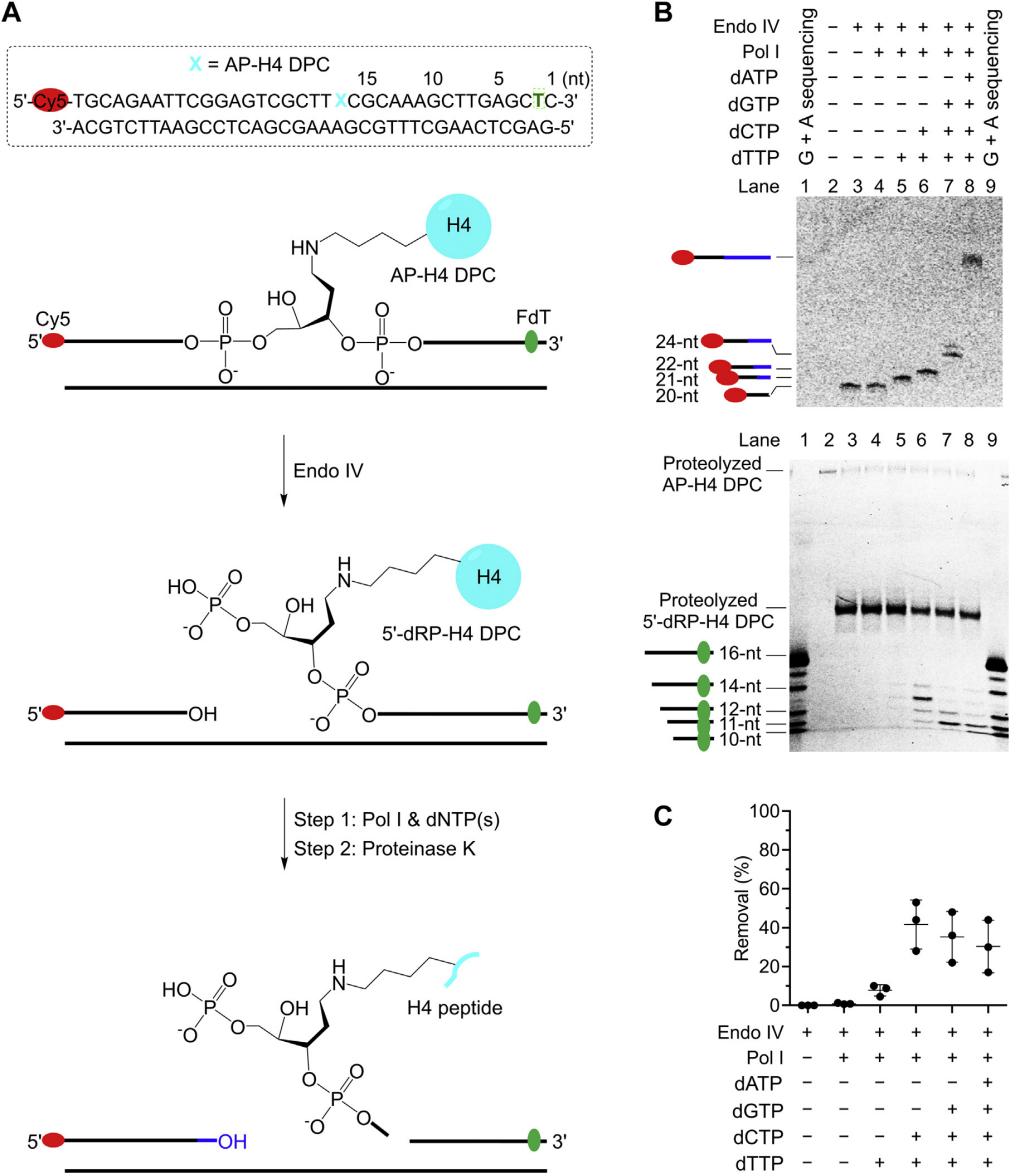
**Pol I removes the 5′-dRP-H4 DPC following strand-displacement DNA synthesis**. *A*, top, the nucleotide sequence of duplex DNA containing an AP-H4 DPC prepared *via* reductive amination. A Cy5 is at the 5′-terminus, and a FdT is at the second position from the 3′-terminus. The numbers above the nucleotide sequence indicate the lengths from the 3′-terminus. Bottom, a scheme showing the procedures of investigating the 5′-dRP-H4 DPC removal by Pol I. The reaction samples were finally treated by proteinase K for urea-PAGE analysis of the unexcised 5′-dRP-H4 DPC. *B*, a representative 20% urea-PAGE gel showing the strand-displacement DNA synthesis (*top*, Cy5) and removal of 5′-dRP-H4 DPC (20 nM, bottom, FdT) by Pol I (4 nM) in the presence of different dNTPs (2 μM) at 37 ^°^C for 30 min. *C*, a scatter plot with the mean and standard deviation showing the efficiency of 5′-dRP-H4 DPC removal by Pol I from the experiments in B (bottom). The data are from three independent experiments. AP site, apurinic/apyrimidinic or abasic site; DPC, DNA–protein cross-link; FdT, fluorescein dT; Pol I, DNA polymerase I.

Having demonstrated that Endo IV efficiently incised the AP-H4 DPC, we asked whether Pol I can subsequently remove the 5′-dRP-H4 DPC. And if so, how is that compared to excising 5′-dRP-peptide_10mer._ Our initial attempt to independently synthesize the 5′-dRP-H4 DPC *via* reductive amination (44) failed likely due to the intrinsic instability of 5′-dRP. Therefore, we prepared the nicked DNA containing 5′-dRP-H4 DPC by incising the AP-H4 DPC with Endo IV (Fig. 11*A*). The AP-H4 DPC containing 5′-Cy5 and FdT at the second position from the 3′-terminus was prepared *via* reductive amination (Fig. S13). During this process, the Cy5 fluorophore was found to be reduced and bleached significantly (data not shown). Therefore, to detect the strand-displacement DNA synthesis products, 20-fold more substrates were used, but the concentrations of all components and reaction conditions remained the same as compared to that in Figure 5 involving the AP-peptide_10mer_. Following nearly complete (∼95%) Endo IV incision of the AP-H4 DPC (Fig. 11*B*, bottom, Lane 3), similar to 5′-dRP-peptide_10mer_ (Fig. 5*C*, bottom), the strand-displacement DNA synthesis by Pol I encountering the 5′-dRP-H4 DPC at all conditions is complete (Fig. 11*B*, top). Notably, 5′-dRP-H4 DPC was removed by Pol I in the presence of dNTPs; however, the maximal efficiency is ∼2-fold lower than that of 5′-dRP-peptide_10mer_ (Figs. 5*D* and 11*C*). These results with a model DPC suggest that proteolysis will facilitate the excision of 5′-dRP-protein DPCs by Pol I.

## Discussion

**Figure 12:**
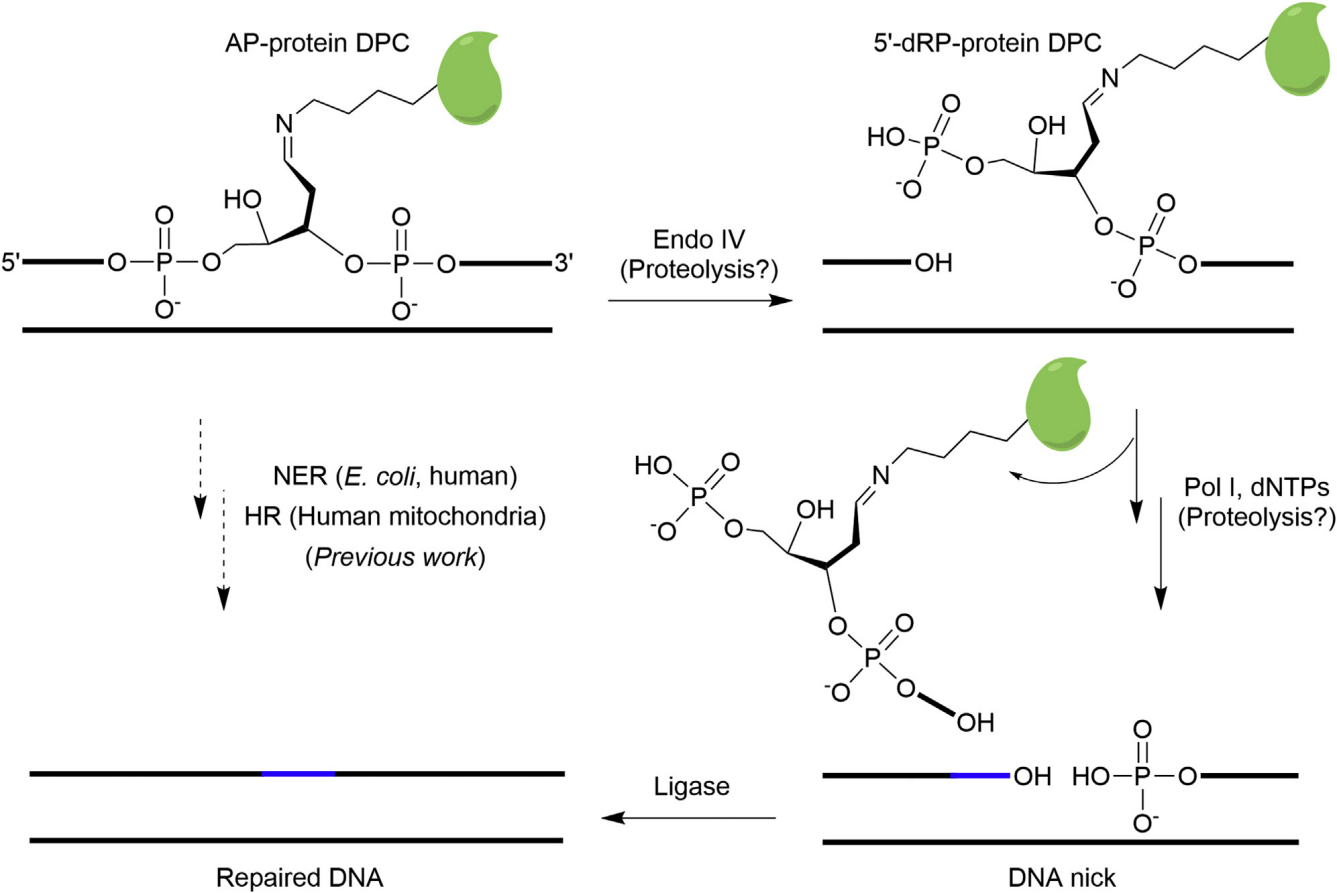
**Proposed repair models of Schiff base AP-protein DPCs**. Previous work has demonstrated that reduced Schiff base AP-protein DPCs can be repaired by *E. coli* and human NER and HR in human mitochondria. In this study, we identified that Schiff base AP-protein DPCs can be repaired by *E. coli* long-patch BER *in vitro*, and proteolysis will likely facilitate the DPC repair. AP site, apurinic/apyrimidinic or abasic site; DPC, DNA–protein cross-link; HR, homologous recombination; NER, nucleotide excision repair.

An AP site is one of the most abundant endogenous DNA lesions (2). It acts as an electrophile that can react with protein nucleophiles (*e.g.,* lysine and cysteine residues) to yield various types of covalent DPCs including Schiff base (16, 17, 18, 22, 24, 25, 45, 46), thiazolidine (47, 48, 49, 50), *S*-glycosidic (51), and *N*-glycosidic (52, 53) bond-linked AP-protein adducts. These DPCs are either new types of DNA lesions or proposed to temporarily protect the lesions from the error-prone repair (17, 49). This study focused on addressing how Schiff base AP-protein DPCs are repaired. These DPCs are unstable but can be long-lived. They need to be removed because they block DNA replication which will greatly threaten the genome integrity (27). This type of DPC is known to be excised by recombinant *E. coli* UvrABC endonuclease and repaired by NER in human cells and HR in human mitochondria likely coupled with DPC proteolysis (28, 29, 30, 31, 32). In this study, we synthesized Schiff base AP-peptide and AP-protein DPC analogs by oxime ligation and reductive amination, respectively, which were used for *in vitro* reconstitution resulting in the discovery and detailed characterization of *E. coli* long-patch BER of Schiff base AP-protein DPCs. This repair process requires a minimum of 3 *E. coli* enzymes and five steps (Fig. 12): (1) 5′-strand incision by Endo IV; (2 to 4) strand-displacement DNA synthesis, removal of the 5′-dRP-peptide/protein adduct-containing flap, and gap-filling DNA synthesis by Pol I; (5) strand ligation by ligase.

Exo III and Endo IV are the two AP endonucleases in *E. coli* cells that incise the AP site at the 5′-side to initiate the repair (Fig. 2), and Exo III accounts for 80% to 90% of the total AP endonucleolytic activity (54, 55). In this study, we demonstrated that Endo IV, but not Exo III, is able to incise AP-peptide/protein adducts, which revealed a possible novel role of this minor AP endonuclease in DNA damage response. Notably, Endo IV plays a major role in incising the AP-peptide adduct within *E. coli* cell extracts. The reason that conjugating a 10-mer peptide, or even a histone H4 (11.2 kDa), to the AP site does not significantly inhibit Endo IV’s activity warrants future investigation.

Pol I excised a 5′-dRP-peptide_10mer_ or 5′-dRP-H4 adduct only when the adduct was located within a flap yielded by strand-displacement DNA synthesis (Figs. 5, 6 and 11), which is possibly due to the reduced steric hindrance. Our results also indicated that the predominant flap excision site of Pol I is at the second nucleotide after the single-strand and double-strand junctions (Figs. 5, 6 and 11). This is distinct from the previous observation that Pol I cuts the strand at the first nucleotide after the junctions to remove a native polynucleotide flap (40). Such discrepancy could be caused by different DNA sequences and/or substrates (native DNA *versus* 5′-dRP-peptide/protein adduct) used in these studies.

Our study demonstrated that removal of AP-protein DPCs by *E. coli* long-patch BER is less efficient than that of an AP-peptide_10mer_ adduct, which agrees with the emerging model that DPC proteolysis by the proteasome or specific DPC proteases is required for efficient DPC repair (56). Since 2014, several proteases have been identified in both yeast and human that are dedicated to DPC proteolysis repair (56). Whether a DPC protease(s) exits in *E. coli* warrants future investigation.

We speculate that the long-patch BER of Schiff base AP-protein DPCs is conserved in prokaryotes and lower eukaryotes (*e.g., Saccharomyces cerevisiae*) due to the presence of corresponding repair enzymes (57, 58). For example, in yeast, the major AP endonuclease, Apn1, is the homolog of Endo IV. The dual function of Pol I in AP-protein DPC removal is likely split into two types of enzymes: strand-displacement DNA synthesis by a DNA polymerase (*e.g*., polymerase δ) and 5′-dRP-peptide/protein removal by a flap endonuclease (*e.g*., Rad27) (57).

## Experimental procedures

### Materials and general methods

All synthetic oligos were purchased from Integrated DNA Technologies and purified by 20% urea-PAGE. Proteinase K (Cat. #: P8107S), Endo IV (Cat. #: M0304S), Exo III (Cat. #: M0206S), Pol I (Cat. #: M0209S), ligase (Cat. #: M0205S), human APE1 (Cat. #: M0282S), uracil-DNA glycosylase (UDG, Cat. #: M0280S), and Nt.BstNBI (Cat. #: R0607S) were purchased from the New England Biolabs. Chemicals were purchased from Sigma-Aldrich and Fisher Scientific. The fluorophore-containing oligos were visualized by a Typhoon 9500 imager. Human histone H4 was purified as previously described (59). pET30a-AlkB was a gift from Tao Pan (Addgene plasmid # 79050) (60). pET30-2-GAPDH was a gift from David Sabatini (Addgene plasmid # 83910) (61). All urea and SDS-PAGE gels were run at room temperature unless otherwise indicated.

### Synthesis of AP-peptide_10mer_ cross-links by oxime ligation

The 10-mer OxyLys-peptide (NH_2_-SGRGXGGKGL-COOH, X is OxyLys) was synthesized by solid-phase peptide synthesis (35). A reaction mixture (100 μl) with dU-containing oligo (Table 1, O2 or O4, 3 nmol), 1 x reaction buffer (20 mM Hepes, pH 7.5, 1 mM DTT), and UDG (final concentration = 0.25 unit/μl) was incubated at 37 °C for 1.5 h. After that, neutralized OxyLys-peptide_10mer_ (43 μl, 7 mM stock) was added to a final concentration of 2 mM, followed by further incubation at 37 °C for 2 h. The reaction mixture was subjected to ethanol precipitation. The residue was resuspended in a Hepes buffer (25 μl, 50 mM, pH 7.5) and heated at 70 °C for 1 h. The heated sample was mixed with an equal volume of loading buffer (85% formamide, 80 mM EDTA) and then purified by a 20% urea-PAGE gel. The desired band was cut, smashed, mixed with an elution buffer (0.2 M NaCl, 1 mM EDTA, 3 ml), and rotated at room temperature overnight. The eluted sample was briefly spun down, and the supernatant was carefully collected, followed by desalting with a 1 ml Sep-Pak C18 cartridge, dried in a speed vacuum, and resuspended in H_2_O (100 μl). The AP-peptide_10mer_ (∼1.5 nmol) was characterized by MALDI-TOF mass spectrometry using 3-hydroxypicolinic acid as the matrix, aliquoted, and stored at −80 °C.

### Synthesis of 5′-dRP-peptide_10mer_ cross-links by oxime ligation

A mixture (100 μl) with a dU-containing oligo (Table 1, O4, O10, or O12, 7.2 nmol), the complementary strand (Table 1, O5, O11, or O13, 11.1 nmol), and 1x buffer (20 mM Hepes, pH 7.5, 100 mM NaCl) was heated at 90 °C for 3 min and then cooled down to room temperature overnight. To convert the dU to an AP site, a reaction mixture (225 μl) containing the above hybridized DNA duplex (6.48 nmol), 1 x buffer (20 mM Hepes, pH 7.5, 1 mM DTT), and UDG (56.3 units) was incubated at 37 °C for 1.5 h. To convert the AP site to 5′-dRP, the above mixture containing the AP site was mixed with APE1 (112.5 units) and 1 x buffer (20 mM Hepes, pH 7.5, 50 mM NaCl, 1 mM DTT, 10 mM MgCl_2_). The mixture (450 μl) was incubated at 37 °C for 2 h followed by quenching with EDTA (final concentration = 20 mM). To conjugate OxyLys-containing peptide_10mer_ to 5′-dRP, to the previous mixture, neutralized peptide (1.13 μmol) was added and incubated at 37 °C overnight. The sample was ethanol precipitated. The residue was resuspended in a Hepes buffer (25 μl, 10 mM, pH 7.5), heated at 70 °C for 1 h, mixed with an equal volume of the loading buffer (85% formamide, 80 mM EDTA), heated at 90 °C for 3 min, and analyzed by 20% urea-PAGE. The desired 5′-dRP-peptide_10mer_ (∼2 nmol) was purified and characterized similarly to AP-peptide_10mer_.

### Synthesis of reduced AP-protein DPCs by reductive amination

Human histone H4, *E. coli* AlkB, and human GAPDH were overexpressed and purified following the reported procedures (59, 60, 61). A reaction mixture (2 ml) with an AP site-containing oligo (2 nmol) prepared from the dU-containing oligo (Table 1, O19), human histone H4 (20 nmol), AlkB (20 nmol), or GAPDH (100 nmol), Hepes buffer (10 mM, pH 7.5), and fresh NaBH_3_CN (50 mM for H4 and AlkB, and 10 mM for GAPDH) was incubated at 37 °C for 19 h, followed by addition of fresh NaBH_3_CN to a final concentration of 100 mM and incubating at 37 °C for 6 h. After that, SDS was added to the mixture to a final percentage of 0.1%. The sample was then concentrated down to ∼50 μl using a 3.5 kDa-cut off Amicon-filter at 16 °C. The concentrated sample was phenol–chloroform extracted, ethanol precipitated, mixed with a loading buffer (50 μl, 20 mM Hepes, pH 7.5, 20% glycerol, 0.85% SDS), heated at 90 °C for 10 min, and finally resolved by 15% SDS-PAGE. The desired DPC band was cut, smashed, mixed with a buffer (3 ml, 0.2 M NaCl, 1 mM EDTA, 0.1% SDS), and rotated at room temperature overnight. After that, the mixture was spun down to pellet the gel particles. The supernatant was carefully taken out, concentrated, and exchanged extensively (12 times, 10-fold dilution/time) to a buffer (50 mM Hepes, pH 7.5) using a 10 kDa cut-off Amicon-filter (0.6 ml) at 16 °C. The concentration of the adduct was determined by SDS-PAGE with the 6-FAM fluorescence using oligo O19 as a reference. The final product (∼800 pmol for H4, 20–50 pmol for AlkB and GAPDH) was aliquoted and stored at −80 °C.

### Incision of AP-peptide_10mer_ by Endo IV

To prepare the double-strand DNA containing 5′-6-FAM-AP-peptide_10mer_, a mixture (100 μl) containing O3 (Table 1, 30 pmol) and sodium phosphate (100 mM, pH 7.5) was heated at 90 °C for 5 min, followed by chilling on ice and adding P1 (Table 1, 20 pmol). The mixture was then incubated at room temperature for 2 h. Double-strand DNA containing 3′-6-FAM-AP-peptide_10mer_ was prepared similarly, but O5 and P2 (Table 1) were used. To determine the incision efficiency of AP-peptide_10mer_ by Endo IV, a reaction mixture (5 μl) containing the AP-peptide_10mer_ duplex (20 nM), 1 x Endo IV buffer (50 mM Hepes, pH 7.9, 100 mM NaCl, 10 mM MgCl_2_, and 1 mM DTT), and Endo IV (0–20 nM) was incubated at 37 °C for 30 min before quenching by adding SDS to a final percentage of 0.2% and an equal volume of loading buffer (85% formamide, 80 mM EDTA, 40 μM oligo O1). The samples were heated at 90 °C for 1 min and analyzed by 20% urea-PAGE. To determine the steady-state kinetic constants, typical reactions (10 μl) containing the above 1x Endo IV buffer, hybridized AP-peptide_10mer_ (5–400 nM), and Endo IV (0.2 nM) were incubated at 37 °C for 5 to 10 min. The reactions were quenched and analyzed as described above. The reaction rates were plotted against the concentration of AP-peptide_10mer_ using the Menten-Michaelis equation (*v = V*_max_ [S]/(*K*_m_ +[S])) by Prism 6.0. The *k*_cat_ was calculated by the equation *k*_cat_ = *V*_max_/[E].

### Strand-displacement DNA synthesis and 5′-dRP-peptide_10mer_ removal by Pol I

To generate the duplex DNA containing an AP-peptide_10mer_ with a 5′-Cy5 and FdT, a reaction (150 μl) containing a sodium phosphate buffer (10 mM, pH 7.5) and oligo O7 (45 pmol) was heated at 90 °C for 5 min and then cooled on ice. AP-peptide_10mer_ (P3, 30 pmol) was then added, followed by incubating at room temperature for 2 h. To generate the nick DNA containing 5′-dRP-peptide_10mer_ from AP-peptide_10mer_, a reaction (18 μl) containing the above hybridized AP-peptide_10mer_ (33 nM), 1 x Endo IV buffer (50 mM Hepes, pH 7.9, 100 mM NaCl, 10 mM MgCl_2_, and 1 mM DTT), and Endo IV (20 nM) were incubated at 37 °C for 2 h. To study the strand-displacement DNA synthesis and 5′-dRP-peptide_10mer_ removal, typical reactions (5 μl) containing the above 5′-dRP-peptide_10mer_ (20 nM), 1 x Pol I buffer (50 mM Hepes, pH 7.9, 100 mM NaCl, 10 mM MgCl_2_, and 1 mM DTT), individual or combined dNTPs (2 μM), and Pol I (4 nM) were incubated at 37 °C for 30 min. The reactions were quenched by an equal volume of a loading buffer (85% formamide, 80 mM EDTA, 0.2% SDS, and 40 μM oligo O1). An aliquot (10 μl) was heated at 90 °C for 1 min, followed by analysis with a 20% urea-PAGE gel. The gel was visualized by using the fluorescence of Cy5 or FdT.

### Excision of independently generated 5′-dRP-peptide_10mer_ by Pol I

To generate the nicked DNA containing the independently synthesized 5′-dRP-peptide_10mer_ within different flap lengths, a reaction (96 μl) containing a sodium phosphate buffer (10 mM, pH 7.5), oligo O8 (100 pmol), and oligo O7 or O9 (50 pmol) was heated at 90 °C for 5 min and then cooled down on ice. Single-strand oligo containing 5′-dRP-peptide_10mer_ (P4, P5, or P6, 20 pmol) was then added, followed by incubating at room temperature for 2 h. The completed hybridization of 5′-dRP-peptide_10mer_ was verified by 20% native-PAGE at 4 °C. To investigate the excision of 5′-dRP-peptide_10mer_ by Pol I, typical reactions (15 μl) containing the above hybridized 5′-dRP-peptide_10mer_ (20 nM), 1 x Endo IV buffer (50 mM Hepes, pH 7.9, 100 mM NaCl, 10 mM MgCl_2_, and 1 mM DTT), and Endo IV (0–20 nM) were incubated at 37 °C for 30 min. The reactions were quenched by an equal volume of a loading buffer (85% formamide, 80 mM EDTA, 0.2% SDS, and 40 μM oligo O1). An aliquot (10 μl) was heated at 90 °C for 1 min, followed by analysis with 20% urea-PAGE.

### Construction of plasmid pHha10-AP-peptide_10mer_

To prepare the 5′-phosphorylated AP-peptide_10mer_ (Table 1, P7), a reaction (80 μl) containing the oligo O17 (Table 1, 5 nmol), 1x buffer (50 mM Tris-HCl, pH 7.5, 10 mM MgCl_2_, 4 mM ATP, 10 mM DTT), and T4 PNK (50 units) was incubated at 37 °C for 4 h. To the reaction mixture, H_2_O (107.5 μl), a Hepes buffer (200 mM stock, pH 7.5, 25 μl), DTT (10 mM stock, 25 μl), and UDG (5 unit/μl stock, 12.5 μl) was sequentially added. The sample (250 μl) was incubated at 37 °C for 1.5 h, followed by phenol–chloroform extraction (2 times) and ethanol precipitation (3 times). The oligo was resuspended in H_2_O (50 μl) and then mixed with the 10-mer OxyLys peptide (250 nmol) in a reaction (67 μl) containing a Hepes buffer (25 mM, pH 7.5). The reaction mixture was incubated at 37 °C overnight. The adduct (1.7 nmol) was purified by 20% urea-PAGE as previously described. The purity and identity were confirmed by MALDI-TOF mass spectrometry and 20% urea-PAGE with SYBR Gold (ThermoFisher, Cat. #: S11494, 10000X) staining. The gap plasmid was produced from pHha10 (42) following a detailed protocol (62). To generate the plasmid (pHha10-AP-peptide_10mer_) containing the above AP-peptide_10mer_ adduct, a reaction (120 μl) containing a buffer (50 mM Tris-HCl, pH 7.5, 10 mM MgCl_2_, 1 mM ATP, 10 mM DTT) and gapped plasmid (2 μg) was heated at 90 °C for 5 min, followed by cooling down on ice and adding the 5′-phosphorylated AP-peptide_10mer_ (7 pmol) prepared above. The mixture (120 μl) was incubated at room temperature for 3 h, followed by sequentially adding H_2_O (42 μl), a buffer (8 μl, 500 mM Tris-HCl, pH 7.5, 100 mM MgCl_2_, 10 mM ATP, 100 mM DTT), ATP (20 μl, 10 mM stock), and T4 DNA ligase (400 units/μl stock, 10 μl). The mixture (200 μl) was incubated at 16 °C for 18 h, followed by heat inactivation (65 °C, 10 min). To remove the unligated plasmid, the above mixture was combined with H_2_O (34.3 μl), a buffer (26.4 μl, 100 mM Bis-Tris-Propane-HCl, 100 mM MgCl_2_, 10 mM DTT), and Exo III (100 units/μl stock, 3.3 μl), followed by incubation at 37 °C for 2 h. The sample was then subjected to purification with a QIAquick PCR Purification Kit (Qiagen, Cat. #: 28104) following the recommended protocol. The final product (550 ng) was stored at −20 °C.

### Reconstitution of *E. coli* long-patch BER of AP-peptide_10mer_

A typical reaction (5 μl) containing a Hepes buffer (50 mM Hepes, pH 7.9, 100 mM NaCl, 10 mM MgCl_2_, and 1 mM DTT), plasmid pHha10-AP-peptide_10mer_ (25 ng), and Endo IV (final conc. = 10 nM) was incubated at 37 °C for 1 h. To the mixture, the above Hepes buffer (0.6 μl), four dNTPs (20 μM stock, 0.7 μl), and Pol I (100 nM stock, 0.7 μl, final conc. = 10 nM) were added. The mixture was then incubated at 37 °C for 1 h. After that, the mixture (7 μl) was combined with a buffer (0.9 μl, 500 mM Tris-HCl, pH 7.5, 100 mM MgCl_2_, 10 mM ATP, 100 mM DTT), and *E. coli* ligase (164 nM stock, 1.1 μl, final conc. = 20 nM). The sample (9 μl) was incubated at 37 °C for 3 h, followed by analysis with a 1% agarose gel supplied with 0.7 X SYBR Gold.

### Strand incision of AP-peptide_10mer_ by *E. coli* cell extracts

*E. coli* strains (Table S1) were obtained from National BioResource Project (NIG, Japan). Cell culture and cell extract preparation were performed following a reported protocol (63). To investigate the strand incision of AP-peptide_10mer_, a reaction mixture (11 μl) containing 1 x buffer (100 mM Tris-HCl, pH 7.5, 5 mM MgCl_2_, 1 mM DTT), ATP (2 mM), β-nicotinamide adenine dinucleotide (0.5 mM), phosphocreatine (5 mM), phosphocreatine kinase (0.2 unit/μl), pHha10-AP-peptide_10mer_ (5 ng/μl), and cell extract (0.2 mg/ml) was incubated at 37 °C. An aliquot (5 μl) was taken out after 5- or 10-min incubation and quenched by adding EDTA (final concentration = 20 mM) and then heating at 70 °C for 3 min. RNase A (80 μg/ml) was then added and incubated at 37 °C for 10 min. After that, SDS (final percentage = 0.5%) and proteinase K (0.8 unit) were added, and the mixture was incubated at 37 °C for 30 min, followed by analysis with a 1% agarose gel supplied with 0.7 X SYBR Gold.

### Incision of AP-protein DPCs by Endo IV

To prepare the double-strand DNA containing AP-protein DPCs, typical reactions (28 μl) containing a sodium phosphate buffer (10 mM, pH 7.5, 100 mM NaCl) and the complementary strand (Table 1, O20, 8.4 pmol) was heated at 90 °C for 5 min, followed by cooling down on ice and adding the AP-protein DPC (5.5 pmol) isolated as described above. The mixture was incubated at room temperature for 2 h. To investigate the incision of AP-protein DPCs by Endo IV, typical reactions (5 μl) containing a buffer (50 mM Hepes, pH 7.9, 100 mM NaCl, 10 mM MgCl_2_, and 1 mM DTT), the hybridized AP-protein DPC (20 nM), and increasing concentrations of Endo IV (0–5 nM) were incubated at 37 °C for 30 min. The reactions were quenched by adding SDS to a final percentage of 0.1%, treated with proteinase K (0.4 unit) at room temperature for 30 min, followed by mixing with an equal volume of a loading buffer (85% formamide, 80 mM EDTA, 0.2% SDS, 40 μM O1) and analyzing by 20% urea-PAGE.

### Strand-displacement DNA synthesis and 5′-dRP-H4 removal by Pol I

To prepare the double-strand DNA containing AP-H4 DPC bearing a 5′-Cy5 and FdT, typical reactions (80 μl) containing a buffer (10 mM sodium phosphate, pH 7.5, 100 mM NaCl) and the complementary strand (Table 1, O7, 600 pmol) were heated at 90 °C for 5 min, followed by cooling down on ice and adding the AP-H4 DPC (400 pmol). The mixture was incubated at room temperature for 2 h. To generate the nicked DNA containing 5′-dRP-H4 from AP-H4, typical reactions (300 μl) containing the above hybridized AP-H4 DPC (33 nM), 1 x Endo IV buffer (50 mM Hepes, pH 7.9, 100 mM NaCl, 10 mM MgCl_2_, and 1 mM DTT) and Endo IV (20 nM) were incubated at 37 °C for 2 h. To study the strand-displacement DNA synthesis and 5′-dRP-H4 removal by Pol I, typical reactions (100 μl) containing the above 5′-dRP-H4 (20 nM) yielded by Endo IV incision, 1 x Pol I buffer (50 mM Hepes, pH 7.9, 100 mM NaCl, 10 mM MgCl_2_, and 1 mM DTT), individual or combined dNTPs (2 μM), and Pol I (4 nM) were incubated at 37 °C for 30 min. The reactions were quenched by addition of SDS to a final percentage of 0.1%, followed by treatment with proteinase K (0.8 units) at room temperature for 30 min. To determine the 5′-dRP-H4 removal, an aliquot of the sample (5 μl, 90 fmol) was mixed with an equal volume of a loading buffer (85% formamide, 80 mM EDTA, 0.2% SDS, and 40 μM oligo O1), heated at 90 °C for 1 min, followed by analysis with 20% urea-PAGE and visualization by using the fluorescence of FdT. To determine the strand-displacement DNA synthesis, the rest of the sample (105 μl, 1.9 pmol) was mixed with the oligo O1 (600 pmol), followed by ethanol precipitation. The precipitated sample was resuspended in a loading buffer (10 μl, 43% formamide, 40 mM EDTA, 0.1% SDS), heated at 90 °C for 1 min, followed by analysis with 20% urea-PAGE and visualization by using the fluorescence of Cy5.

## Data availability

All data are contained within the article and supporting information.

## Supporting information

This article contains supporting information.

## Conflict of interest

The authors declare that they have no conflicts of interest with the contents of this article.

## Supporting information

Bryan_JBC_SI_Revised_05.18.22
