# Supplementary material for: In vitro eradication of abasic site-mediated DNA–peptide/protein cross-links by Escherichia coli long-patch base excision repair
Source: J Biol Chem. 2022 May 20;298(7):102055. doi: 10.1016/j.jbc.2022.102055 (PMC9234237; doi:10.1016/j.jbc.2022.102055)
Supplement: Bryan_JBC_SI_Revised_05.18.22 [file mmc1.pdf]

Supporting Information

In vitro eradication of abasic site-mediated DNA-peptide/protein cross-links by *Escherichia coli* long-patch base excision repair

Cameron Bryan[1], Xiaoying Wei[1,2], Zhishuo Wang[1], and Kun Yang[1]*

[1] Division of Chemical Biology and Medicinal Chemistry, College of Pharmacy, The University of Texas at Austin, Austin, Texas 78712, United States

[2] Department of Molecular Biosciences, The University of Texas at Austin, Austin, Texas 78712, United States

* To whom correspondence should be addressed. Tel: +1 512-471-4843, Email: kun.yang@austin.utexas.edu

Table S1. *E. coli* strains used in this study.

Source	Strain name	Description
ME collection	BW25113 (ME9062)	Parent (Wild-type) strain
Keio Collection	JW4019-KC	BW25113 + *uvrA* deletion
Keio Collection	JW0762-KC	BW25113 + *uvrB* deletion
Keio Collection	JW1898-KC	BW25113 + *uvrC* deletion
Keio Collection	JW2146-KC	BW25113 + *nfo* deletion

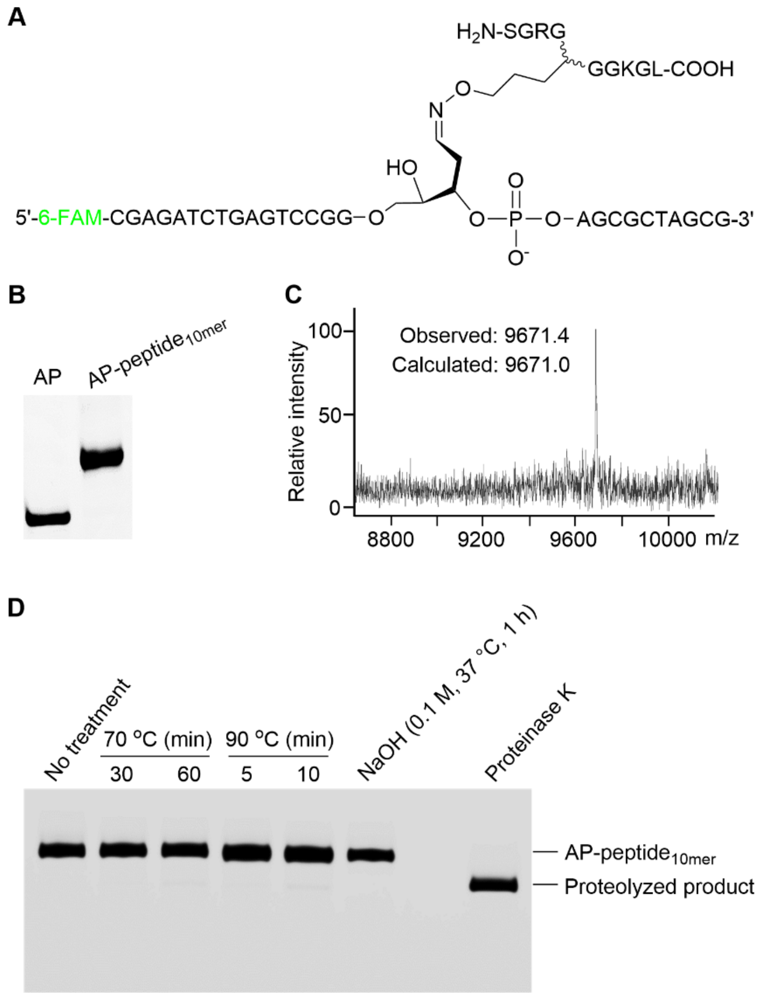

Fig. S1. Characterization of AP-peptide$_{10mer}$. A. The nucleotide sequence and structure of AP-peptide$_{10mer}$. B. A 20% urea-PAGE gel showing the purity of isolated AP-peptide$_{10mer}$. C. MALDI-TOF mass spectrometry characterization of AP-peptide$_{10mer}$. D. A 20% urea-PAGE gel showing the stability of AP-peptide$_{10mer}$ after heating or NaOH treatment. The oligos and adducts were visualized by using the fluorescence of 6-FAM. This cross-link was used for the experiments in Figure 4A, Fig. S3, and Fig. S4.

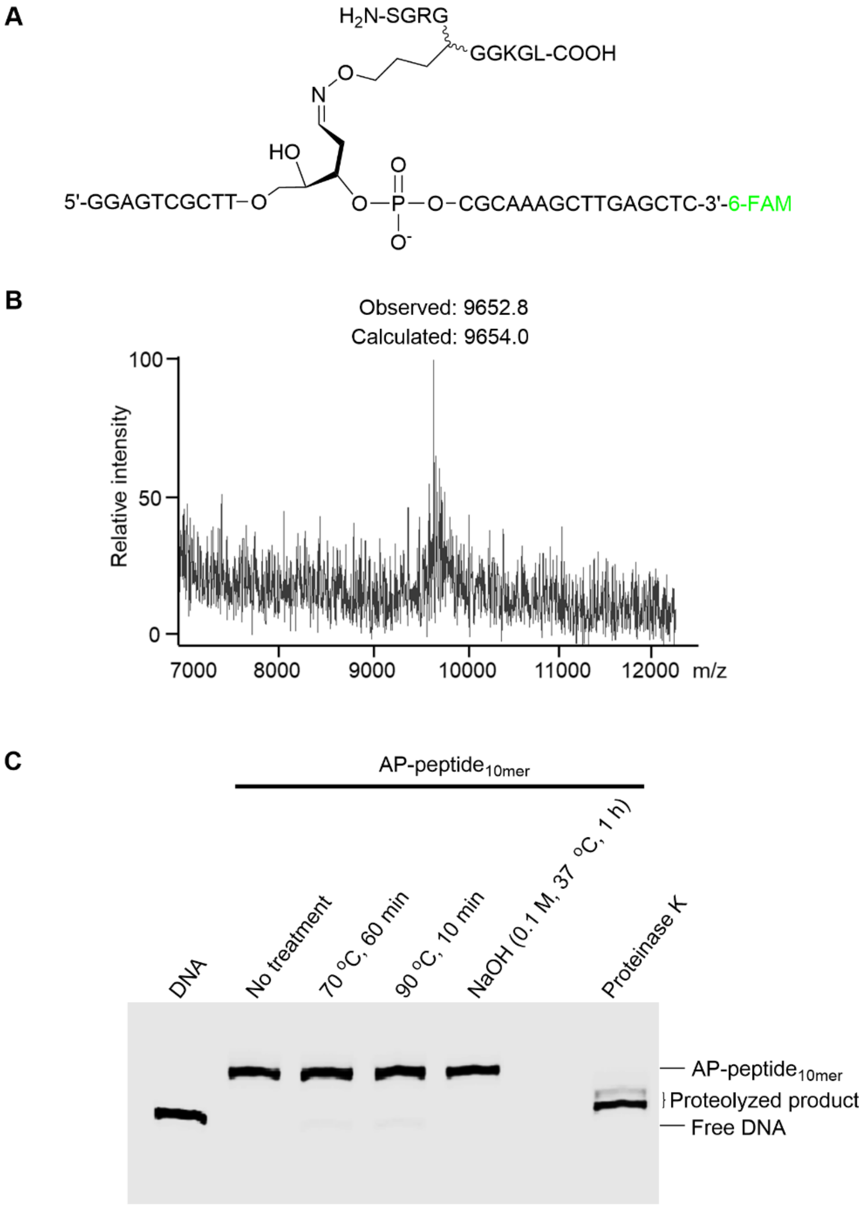

Fig. S2. Characterization of AP-peptide$_{10mer}$. A. Nucleotide sequence and structure of AP-peptide$_{10mer}$. B. MALDI-TOF mass spectrometry characterization of AP-peptide$_{10mer}$. C. A 20% urea-PAGE gel showing the stability of AP-peptide$_{10mer}$ after heating or NaOH treatment. The oligos and adducts were visualized by using the fluorescence of 6-FAM. This cross-link was used for the experiments in Figure 4B.

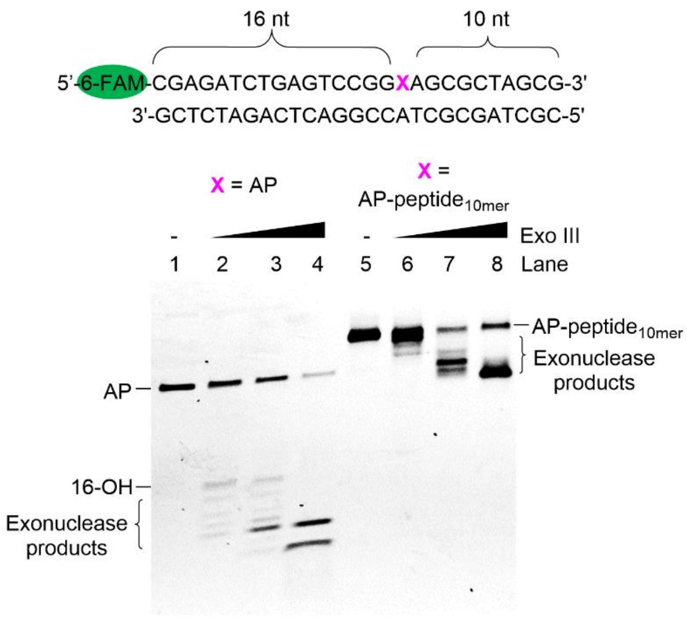

Fig. S3. Exo III incised the AP site, but not AP-peptide$_{10mer}$. The reaction (10 μL) containing the AP site or AP-peptide$_{10mer}$ (20 nM) was incubated with increasing concentration of Exo III (0-500 nM) at 37 ºC for 30 min, followed by analysis with a 20% urea-PAGE gel. The oligos and adducts were visualized by using the fluorescence of 6-FAM.

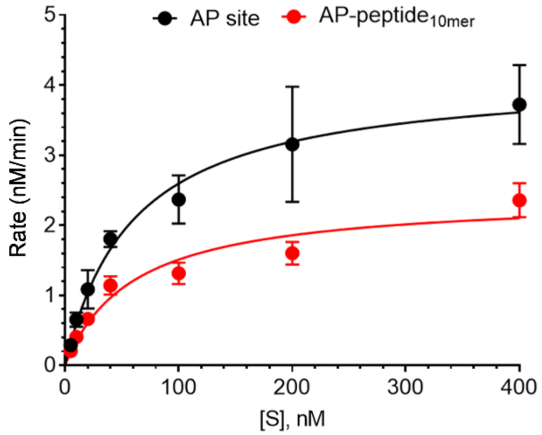

Fig. S4. Michaelis-Menten saturation curves of incising the AP site and AP-peptide$_{10mer}$ by Endo IV. The data is the average and standard deviation from three independent experiments. The kinetic constants were summarized in Table 2.

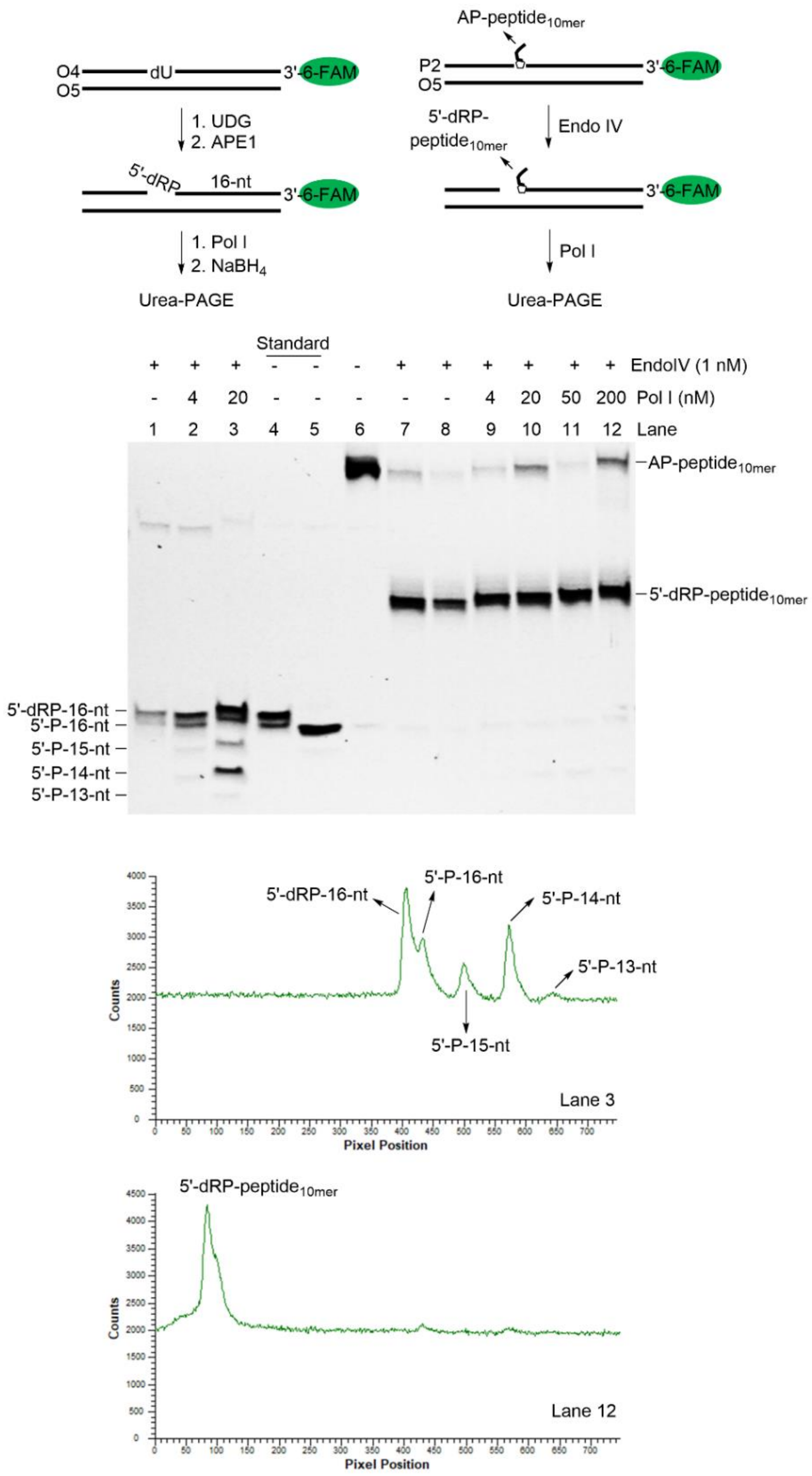

Fig. S5. Pol I excises 5′-dRP but not 5′-dRP-peptide$_{10mer}$. To investigate the removal of 5′-dRP by Pol I, dU-containing duplex DNA (20 nM) was sequentially treated by UDG and APE1, and then Pol I (37 ºC, 30 min). The samples were stabilized by $NaBH_4$ before urea-PAGE (20%) analysis. To investigate the removal of 5′-dRP-peptide$_{10mer}$ by Pol I, the hybridized AP-peptide$_{10mer}$ (20 nM) was sequentially treated by Endo IV and Pol I (37 ºC, 30 min), followed by 20% urea-PAGE analysis. The oligos and adducts were visualized by using the fluorescence of 6-FAM.

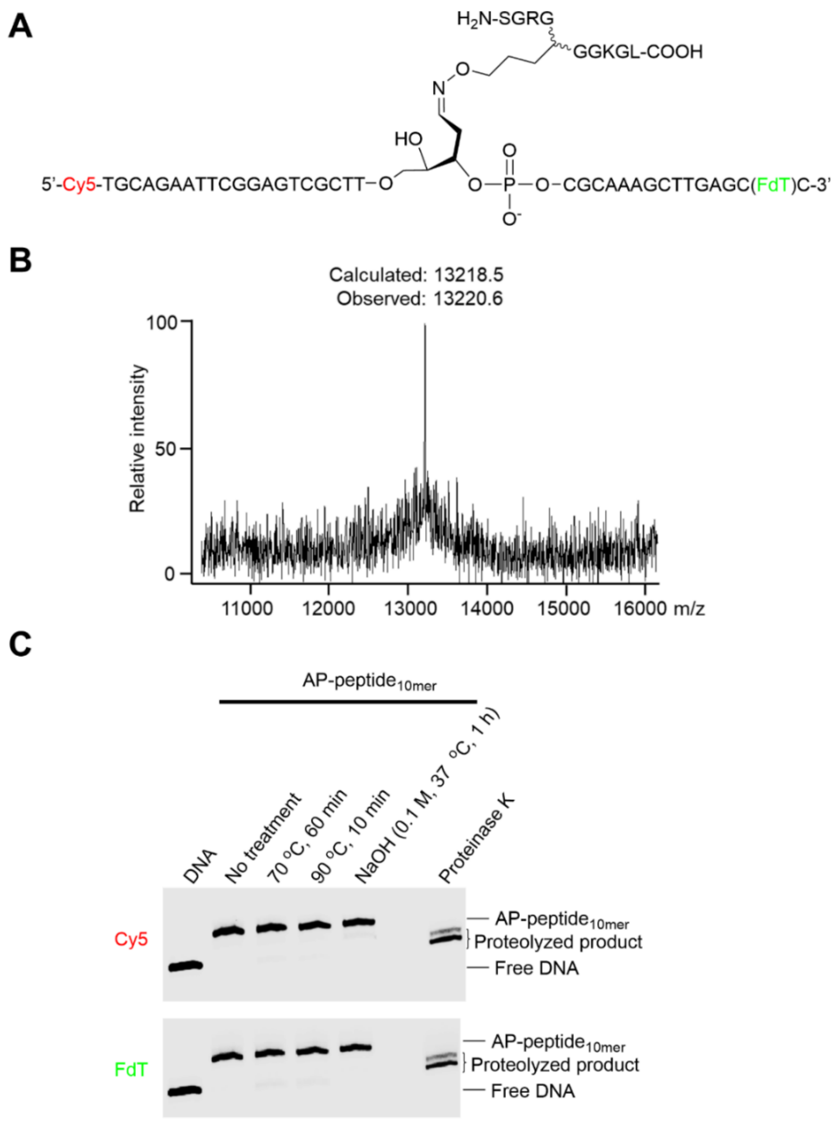

Fig. S6. Characterization of AP-peptide$_{10mer}$. A. The nucleotide sequence and structure of AP-peptide$_{10mer}$. B. MALDI-TOF mass spectrometry characterization of AP-peptide$_{10mer}$. C. A 20% urea-PAGE gel showing the stability of AP-peptide$_{10mer}$ after heating and NaOH treatment. The oligos and adducts were visualized by using the fluorescence of Cy5 or FdT. This cross-link was used for the experiments in Figure 5.

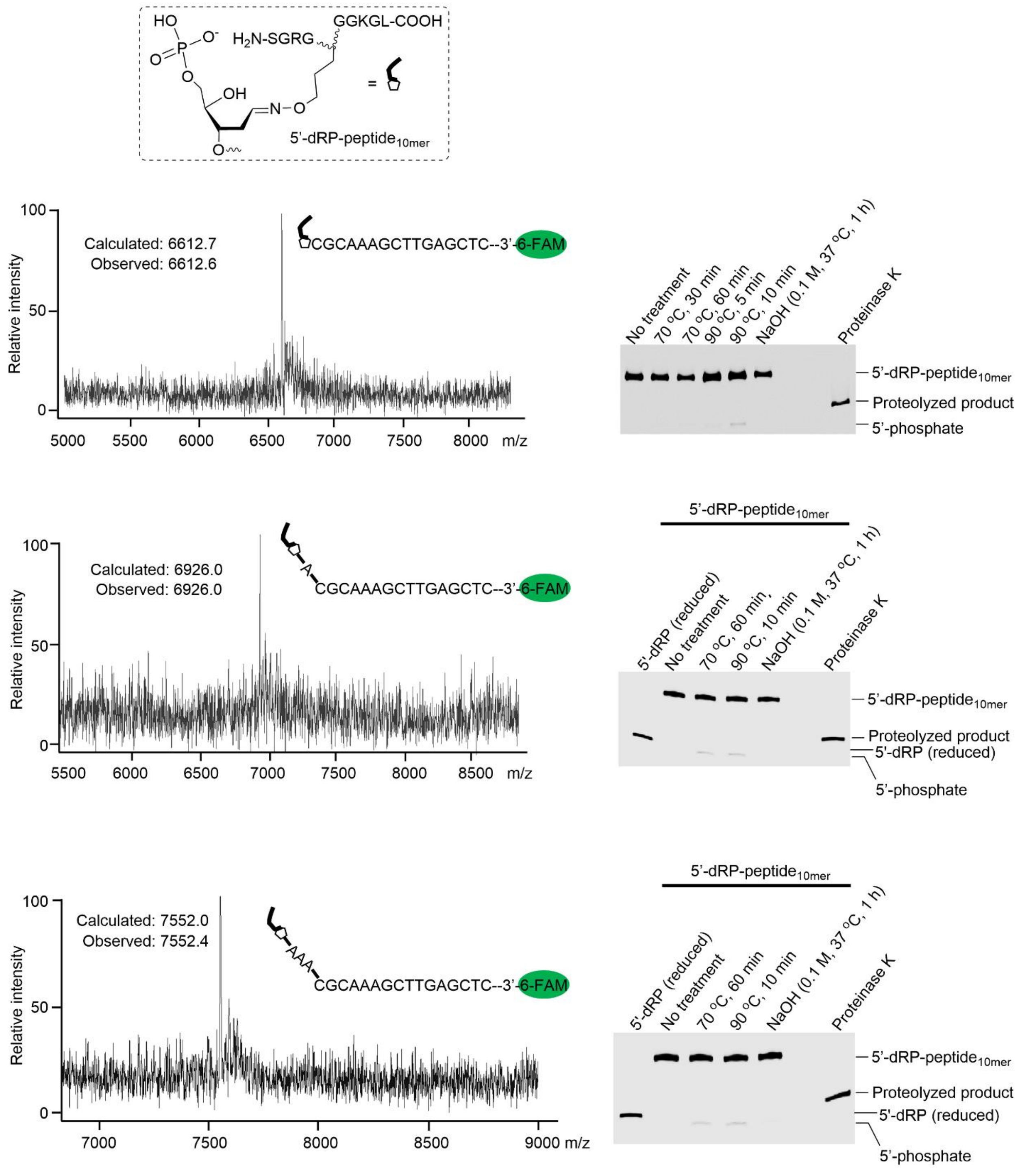

Fig. S7. Characterization of 5′-dRP-peptide$_{10mer}$. Left, the nucleotide sequence, structure, and MALDI-TOF mass spectrometry characterization of 5′-dRP-peptide$_{10mer}$. Right, 20% urea-PAGE gels showing the stability of 5′-dRP-peptide$_{10mer}$ after heating and NaOH treatment. The oligos and adducts were visualized by using the fluorescence of 6-FAM. These cross-links were used for the experiments in Figure 6.

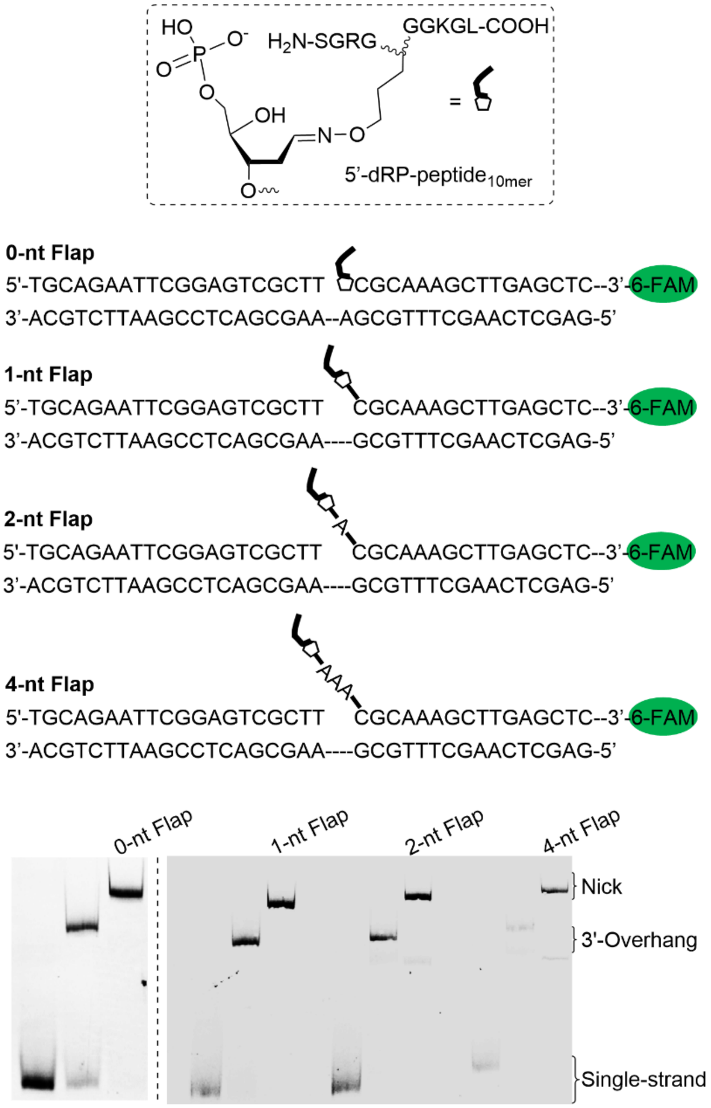

Fig. S8. Sequence, structure, and native PAGE (20%) verification of the hybridized nicked DNA containing 5′-dRP-peptide$_{10mer}$. The adducts were visualized by using the fluorescence of 6-FAM. These substrates were used for the experiments in Figure 6.

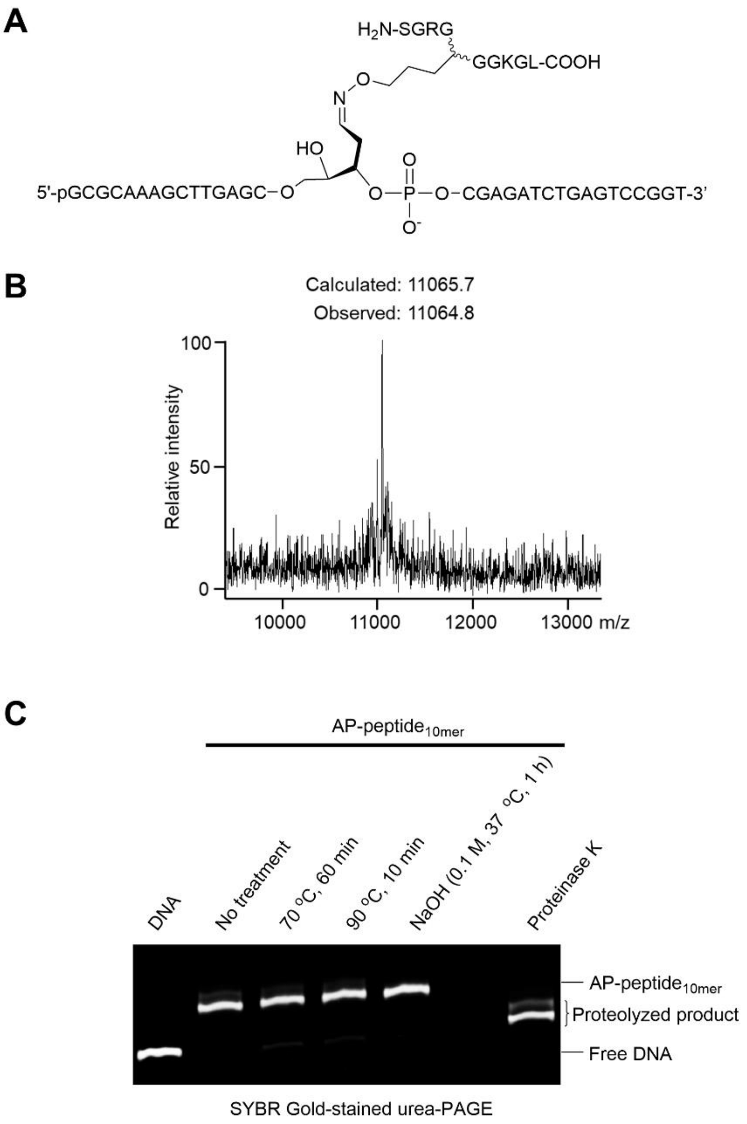

Fig. S9. Characterization of AP-peptide$_{10mer}$. A. The nucleotide sequence and structure of AP-peptide$_{10mer}$. B. MALDI-TOF mass spectrometry characterization of AP-peptide$_{10mer}$. C. A 20% urea-PAGE gel showing the stability of AP-peptide$_{10mer}$ after heating and NaOH treatment. The gel was stained with SYBR Gold at room temperature for 5 min before visualization. This cross-link was used to construct the plasmid pHha10-AP-peptide$_{10mer}$ that was used for the experiments in Figures 8 and 9.

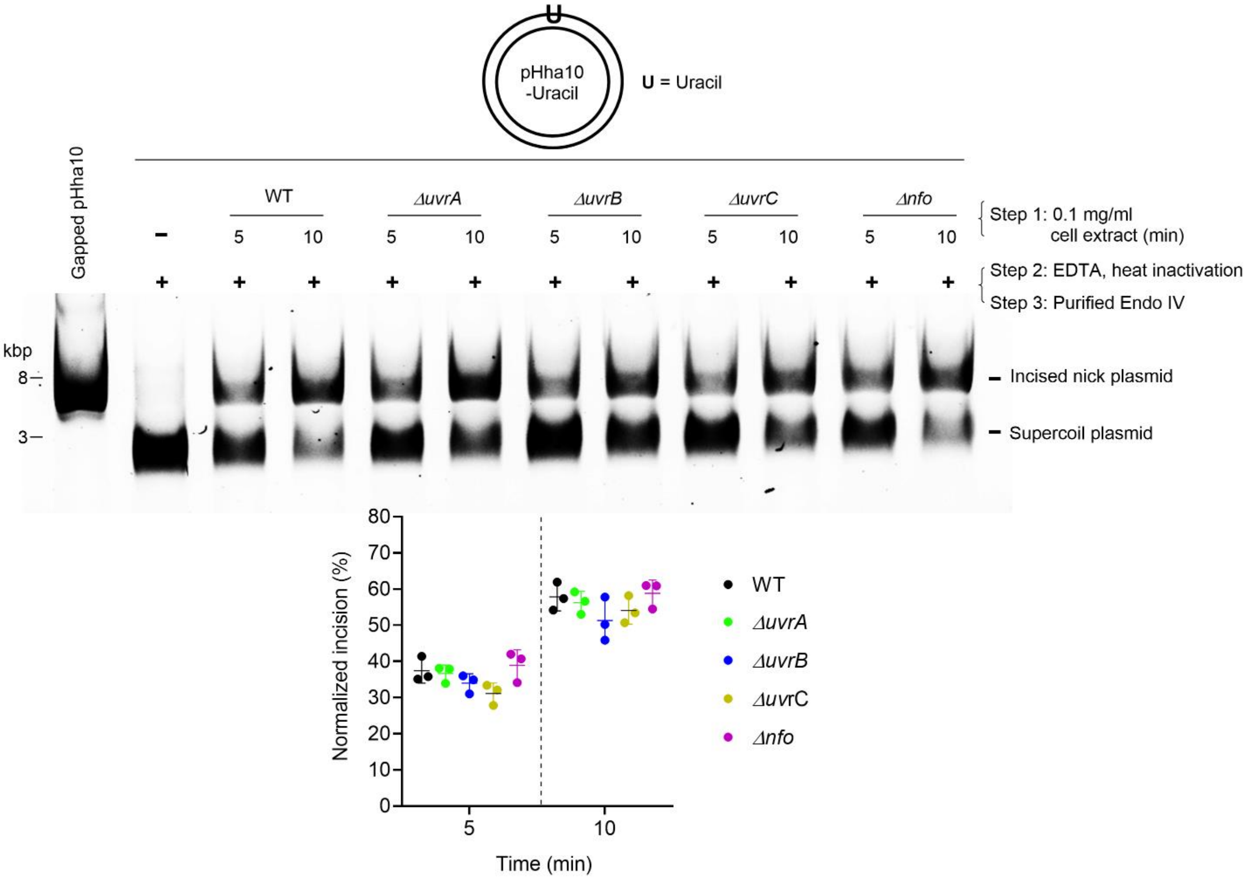

Fig. S10. A control experiment comparing the activity of uracil-DNA glycosylase within different *E. coli* cell extracts. Top, a representative 1% agarose gel (pre-stained with SYBR Gold) analyzing the following reactions. Briefly, an uracil-containing plasmid (pHha10-Uracil, 25 ng) was treated by wild-type (WT) or DNA repair-deficient *E. coli* cell extracts (0.1 mg/mL, 37 ^oC, 5 or 10 min). During this reaction, the uracil will be removed by uracil-DNA glycosylase within the cell extracts to yield an AP site. To ensure that all AP sites are incised to yield nick plasmids for quantification, the previous reactions were first terminated by adding EDTA (20 mM) and heating (70 ^oC, 3 min), and then treated with recombinantly purified Endo IV (4.5 nM, 37 ^oC, 30 min). All samples were finally mixed with SDS (final percentage = 0.5%) and treated with proteinase K (0.8 unit, 37 ^oC, 30 min) before agarose gel analysis. Bottom, a scatter plot with the mean and standard deviation showing the strand incision efficiency of pHha10-Uracil as a function of time. The data are from three independent experiments. Based on these results, we conclude that, compared to other types of cell extracts, the *nfo*-deficient cell extract has similar activity of uracil-DNA glycosylase.

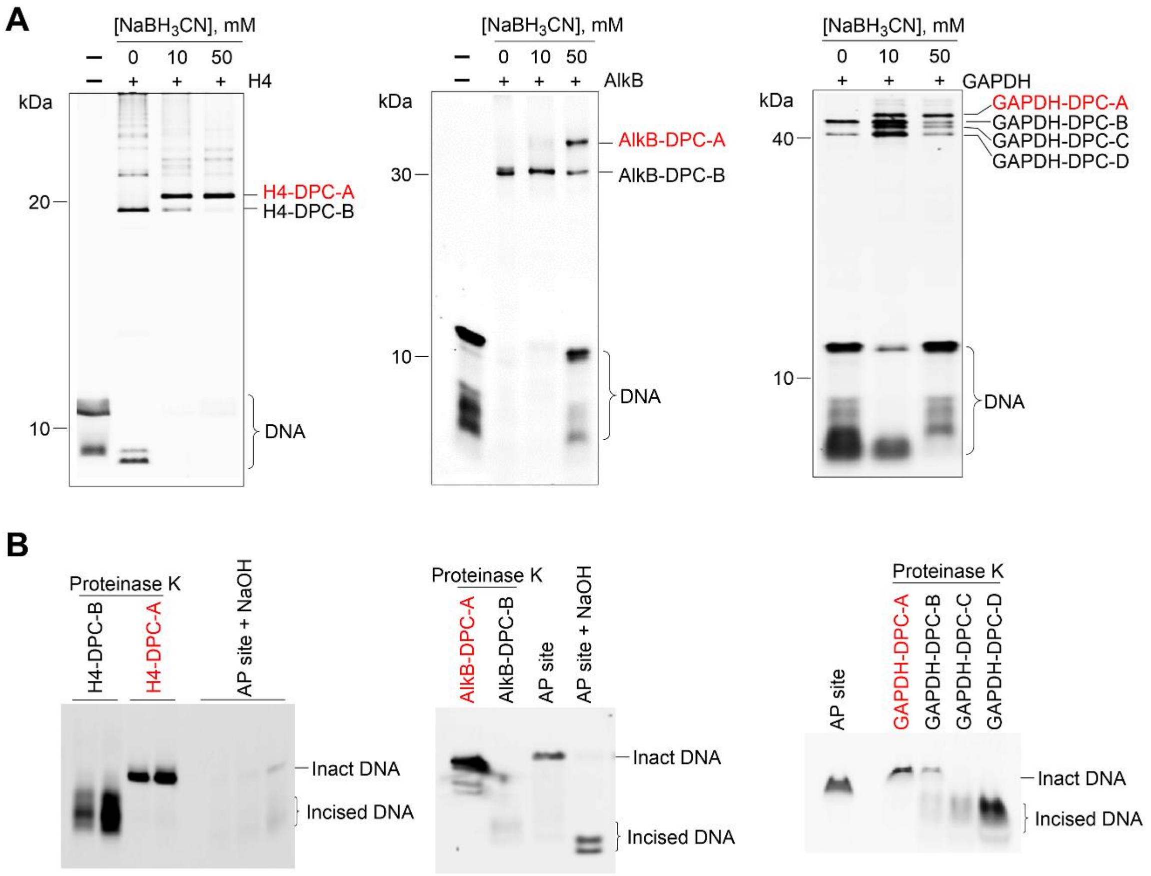

Fig. S11. Preparation and verification of reduced Schiff base AP-protein DPCs. A. 15% SDS-PAGE gels showing the DPC formation following reacting the AP site (prepared from oligo O19) with histone H4, AlkB, or GAPDH. B. 15% SDS-PAGE gels comparing the migration of proteolyzed DPCs with the (NaOH-incised) AP site. H4-DPC-A, AlkB-DPC-A, and GAPDH-DPC-A that were highlighted in red are the desired DPC products because the proteins are cross-linked to the AP site within uncleaved (intact) DNA. The DNA and DPCs were visualized by using the fluorescence of 6-FAM.

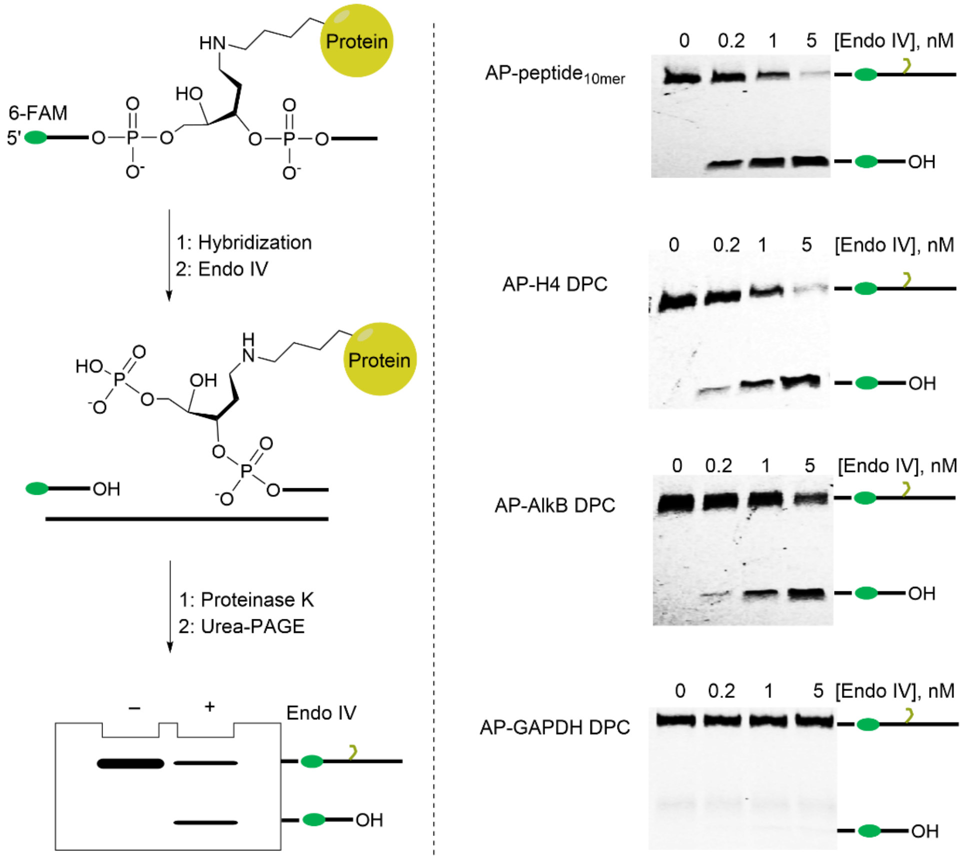

Fig. S12. Incision of AP-protein DPCs by Endo IV. Left, a scheme showing the strand incision of AP-protein DPCs by Endo IV, and the sample treatment before urea-PAGE analysis. To allow the urea-PAGE analysis of the remaining uncleaved AP-protein DPCs, the reaction samples were treated by proteinase K following Endo IV incision. Right, representative 20% urea-PAGE gels showing the strand incision of AP-protein DPCs (20 nM) by Endo IV (0.2, 1, or 5 nM) at 37 °C for 30 min. The quantified data were plotted in Figure 10B. The DNA and proteolyzed DPCs were visualized by using the fluorescence of 6-FAM.

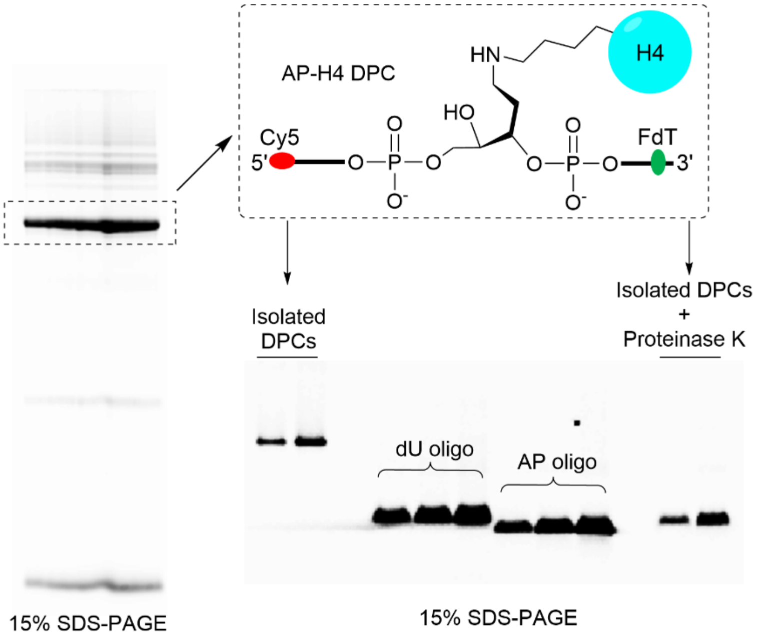

Fig. S13. Preparation of an AP-H4 DPC with 5′-Cy5 and FdT at the second position from the 3′-terminus via reductive amination. 15% SDS-PAGE gels showing the preparation, isolation, and verification of the reduced Schiff base AP-H4 DPC. The DNA and DPCs were visualized by using the fluorescence of FdT. This AP-H4 DPC was used for the experiments in Figure 11.
